# Harnessing Oxidized Alginate Microgels for Rapid and Self‐Assembling Dental Tissue Organogenesis In Vitro and In Vivo

**DOI:** 10.1002/smsc.202500053

**Published:** 2025-10-03

**Authors:** Chao Liang, Shuxuan Wu, Ziqi Huang, Zhenzhen Wu, Siyuan Chen, Feiming Li, Karrie Mei‐Yee Kiang, Gilberto Ka‐Kit Leung, Indong Jun, Hwan D. Kim, Ann‐Na Cho, Hee Jung Lee, Honghyun Park, Yiu Yan Leung, Seong Jun Kim, Seil Sohn, Haram Nah, Jae Seo Lee, Il Keun Kwon, Dong‐Nyoung Heo, Sang‐woo Lee, Zhaoming Wu, Sang Jin Lee

**Affiliations:** ^1^ Biofunctional Materials Division of Applied Oral Sciences and Community Dental Care Faculty of Dentistry The University of Hong Kong 34 Hospital Road Hong Kong SAR 999077 China; ^2^ Craniofacial Development Division of Applied Oral Sciences and Community Dental Care Faculty of Dentistry The University of Hong Kong 34 Hospital Road Hong Kong SAR 999077 China; ^3^ Department of Surgery School of Clinical Medicine LKS Faculty of Medicine The University of Hong Kong Hong Kong Hong Kong SAR 999077 China; ^4^ The State Key Laboratory of Brain and Cognitive Sciences The University of Hong Kong Pokfulam Hong Kong SAR 999077 China; ^5^ Environmental Safety Group Korea Institute of Science & Technology Europe (KIST‐EUROPE) 66123 Saarbrücken Germany; ^6^ Department of Biomedical Sciences Seoul National University of Science and Technology Seoul 01811 Republic of Korea; ^7^ School of Biomedical Engineering Faculty of Engineering The University of Sydney Darlington NSW 2008 Australia; ^8^ Composites Research Division Korea Institute of Materials Science 797 Changwon‐daero, Seongsan‐gu Changwon 51508 Republic of Korea; ^9^ Advanced Bio and Healthcare Materials Research Division Korea Institute of Materials Science 797 Changwon‐daero, Seongsan‐gu Changwon 51508 Republic of Korea; ^10^ Oral and Maxillofacial Surgery, Faculty of Dentistry The University of Hong Kong 34 Hospital Road Hong Kong SAR 999077 China; ^11^ Department of Neurosurgery CHA Bundang Medical Center CHA University Seongnam‐si Gyeonggi‐do 13449 Republic of Korea; ^12^ Department of Dental Materials School of Dentistry Kyung Hee University 26 Kyungheedae‐ro Seoul 02447 Republic of Korea; ^13^ Biofriends Inc 26 Kyungheedae‐Ro Seoul 02447 Republic of Korea; ^14^ Department of Physiology School of Dentistry and Dental Research Institute Seoul National University Seoul 08826 Republic of Korea

**Keywords:** cellular condensation, craniofacial regeneration, dental stem cells, epithelial–mesenchymal interactions, oxidized alginate microgels, tooth development, tooth germs

## Abstract

Regenerating dental tissues for craniofacial reconstruction remains challenging due to inadequate tissue organization and poor intercellular connectivity, often caused by residual biomaterials. Recapitulating key developmental processes, such as spontaneous cellular condensation and epithelial–mesenchymal interactions (EMI), is essential for engineering functional tissue architecture. This study introduces an innovative system that utilizes oxidized alginate (OA) microgels laden with high‐density human dental stem cells to promote self‐condensation and EMI. The OA microgels were prepared through sodium periodate oxidation and further optimized. In vitro studies demonstrated rapid self‐degradation of OA, which promoted efficient cell condensation and robust 3D tissue formation. Following subcutaneous transplantation into mice, the cell‐dense microgels exhibited functional integration with host tissues, along with robust vascularization and osteogenic differentiation. To demonstrate its potential for craniofacial regeneration, a tooth germ model (OA/Epithelium + OA/Mesenchyme) that mimics EMI was developed using embryonic dental epithelial and mesenchymal cells from Embryonic Day 14.5 mice. Immediate transplantation under the mouse kidney capsule resulted in bone organogenesis within two weeks. In summary, the OA microgel system provides initial mechanical support and then quickly degrades to enable critical cell‐cell interactions that mirror organ development. Thus, this scalable and cost‐effective approach holds significant promise for advancing dental tissue engineering.

## Graphical abstract

The system employs a high density human dental stem cells‐laden oxidized alginate‐based microgel with a "degrade‐to‐active" paradigm that mimics cellular condensation to enhance cell viability, achieve robust vascularization, and promote subsequent dental organogenesis, which is enabled by rapid degradation along with the elimination of residual biomaterials that typically hinder tissue engineering.

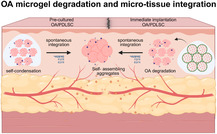

## 1 Introduction

The loss of craniofacial tissue due to disease, trauma, or congenital defects can have a devastating impact on a person's appearance, function, and mental well‐being, necessitating urgent efforts to regenerate damaged tissue.^[^ ^1^ ^]^ Current clinical standards, such as autografts and allografts, often require additional surgical procedures, which carry associated comorbidities and expose patients to a higher risk of immune rejection and infection.^[^ ^2^ ^]^ Over the past few decades, tissue engineering using biological 3D scaffolds has emerged as a promising alternative to promote craniofacial reconstruction.^[^ ^3^, ^4^ ^]^ For instance, bioactive tricalcium phosphate grafts have been shown to promote local bone volumetric increase clinically. In another study, a biocompatible composite scaffold was used to reconstruct tissues in the defected region with enhanced tissue integration.^[^ ^5^ ^]^ However, such acellular scaffolds often exhibit limited cellular affinity, leading to their recognition as foreign bodies and potential immune‐mediated graft failure.^[^ ^2^, ^6^, ^7^ ^]^

To circumvent these issues, scientists and clinicians have focused on developing human‐derived dental stem cells (hDSCs) as a therapeutic approach through direct injection or transplantation in a scaffold‐free manner.^[^ ^8^, ^9^, ^10^ ^]^ Various hDSCs have been shown to have regenerative potential that can be primed through in vitro predifferentiation, enabling them to facilitate new tissue repair in vivo.^[^ ^11^, ^12^ ^]^ Unfortunately, the application of cell suspensions or engineered cell sheets directly into tissue defects often fails to generate new tissues with  intracellular complexity that resembles natural tissues and organs.^[^ ^9^ ^]^ Indeed, assembling individual hDSCs into cohesive cellular aggregates or functional 3D structures remains a major obstacle, and technical hurdles related to producing a readily implantable system persist. One promising strategy is to provide a temporarily supportive niche that enables rapid cell aggregation and is easily removable for immediate implantation.

Significant efforts have been made over the last decade to fabricate 3D dental tissue, particularly using tissue engineering techniques. Notably, self‐assembling technologies have emerged for the fabrication of assembloid models.^[^ ^13^ ^]^ This self‐assembling strategy aims to enable de novo regeneration through self‐condensation,^[^ ^14^ ^]^ which is crucial in craniofacial tissue and tooth development. However, current technologies still struggle to establish a truly fast‐release, scaffold‐less structure, where the key lies in adopting a temporary vehicle for immediately injectable cell encapsulation and rapid cell–cell assembly. Also, self‐assembling strategy has been explored based on the modification of alginate‐based hydrogel since they have been widely used as carriers for hDSC therapy due to their biocompatibility, injectability,^[^ ^11^, ^15^ ^]^ and ability to provide a 3D environment for tissue growth,^[^ ^5^, ^16^ ^]^ promote vascularization,^[^ ^17^, ^18^ ^]^ and suppress inflammation.^[^ ^19^, ^20^ ^]^ Modification is aimed to deal with the concern about the persistence of nondegradable alginate in the body that hinders tissue integration, potentially leading to graft failure.^[^ ^11^ ^]^ While exploring specialized devices, long‐term cultures, and hanging‐drop procedures for washout or alginate enzymes for biodegradation is worthwhile, these methods are labor‐intensive, costly, complicated, and not easily scalable and may compromise the potential of stem cells.^[^ ^21^, ^22^ ^]^ To address these challenges, oxidized alginate (OA) has recently emerged as a material with the desired degradation behavior, holding a promise to facilitate the formation of spontaneously assembling aggregates.^[^ ^15^, ^23^, ^24^ ^]^ Although OA shows promising degradability, spontaneous tissue formation leading to new tissue reconstruction in defect areas remains a significant challenge.^[^ ^25^, ^26^, ^27^ ^]^ There is a critical need for an OA‐based system that actively promotes the high‐density cell‐cell interactions necessary for efficient self‐condensation and organogenesis.

**Figure 1:**
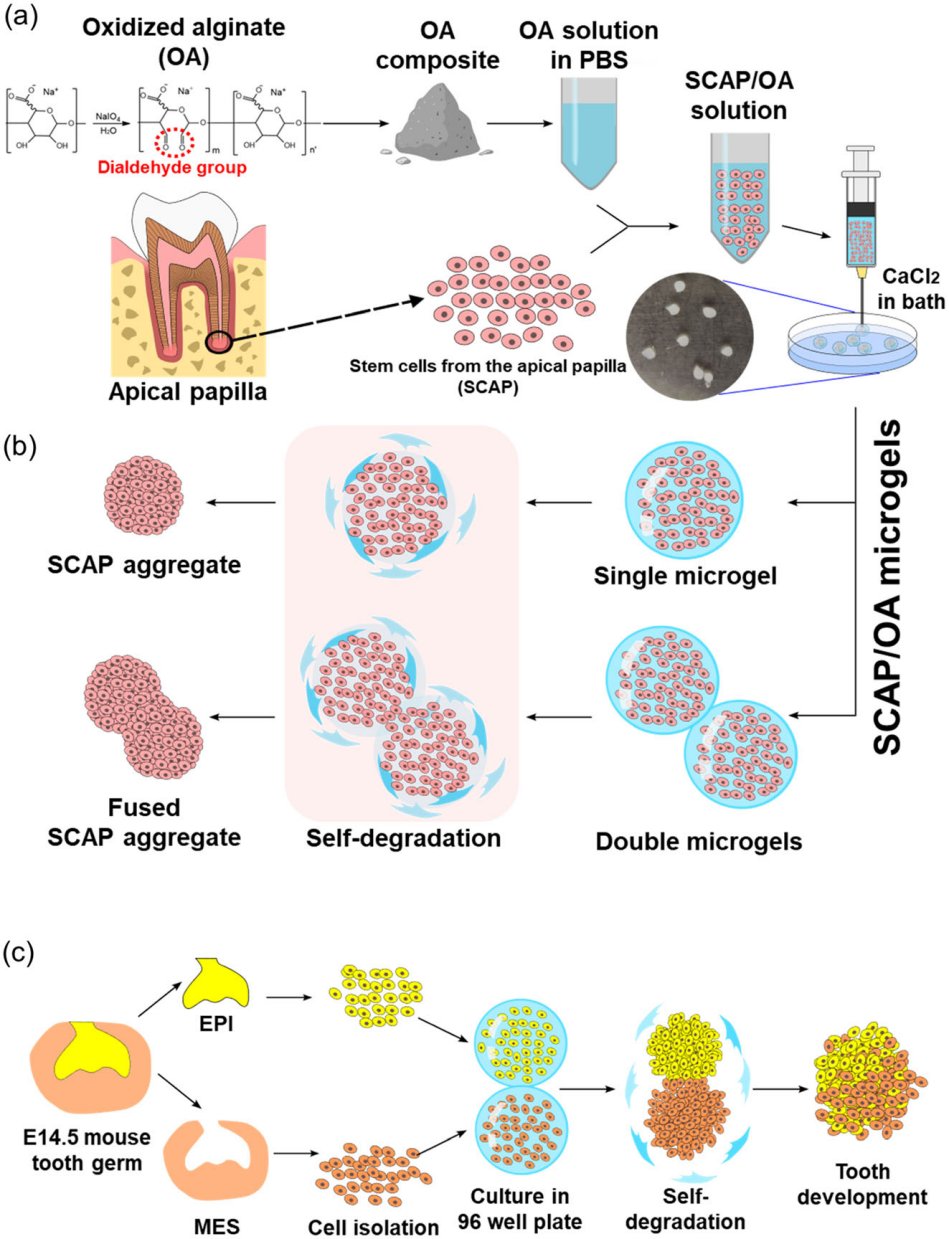
Schematic illustration of high‐density dental cells loaded in 5OA microgels for rapid cellular organogenesis. a) Step 1: fabrication process of 5OA/SCAP microgels using a CaCl_2_ cross‐linker, which initiates the gelation of 5OA to form stable microgel structures. b) Step 2: confirmation of the self‐assembling behavior of individual OA/SCAP microgels, showcasing their ability to spontaneously integrate when two 5OA/SCAP microgels are combined, resulting in a cohesive aggregate. c) Step 3: functional validation of the system using a tooth germ model, illustrating the self‐condensation and spontaneous integration of EPI‐laden 5OA microgels and MES‐laden 5OA microgels. The self‐degradation properties of 5OA facilitate efficient cell condensation and aggregation, promoting the formation of organized tissue structures.

Building on our previous work that regulated OA oxidation levels to promote cell differentiation and tissue integration,^[^ ^28^ ^]^ we now propose a novel strategy: high‐density hDSC‐laden OA microgels promoting cell‐driven condensation for organogenesis. This approach eliminates the need for complex enzymatic or chemical hydrogel removal, streamlining the path to immediate transplantation. To demonstrate the aggregate formation capability, we successfully prepared OA and generated multiple condensates in subsequent in vitro cultures. Our approach describes the self‐condensation and spontaneous integration of hDSCs, such as human stem cells from the apical papilla (SCAP), as well as the formation of a tooth germ model using embryonic dental cells in vitro. Furthermore, this study highlights the ability of released periodontal ligament stem cells (PDLSCs) encapsulated in OA microgels to achieve spontaneous tissue integration in a mouse subcutaneous model. We also confirmed the potential for tissue integration and in vivo self‐organization through the implantation of tooth germ encapsulated in OA microgels into mouse renal capsules. A detailed schematic illustration of our study strategy is provided in **Figure** **1**.

## 2 Results and Discussion

### 2.1 Characterization of Unmodified Alginate and Various OA Conditions

**Figure 2:**
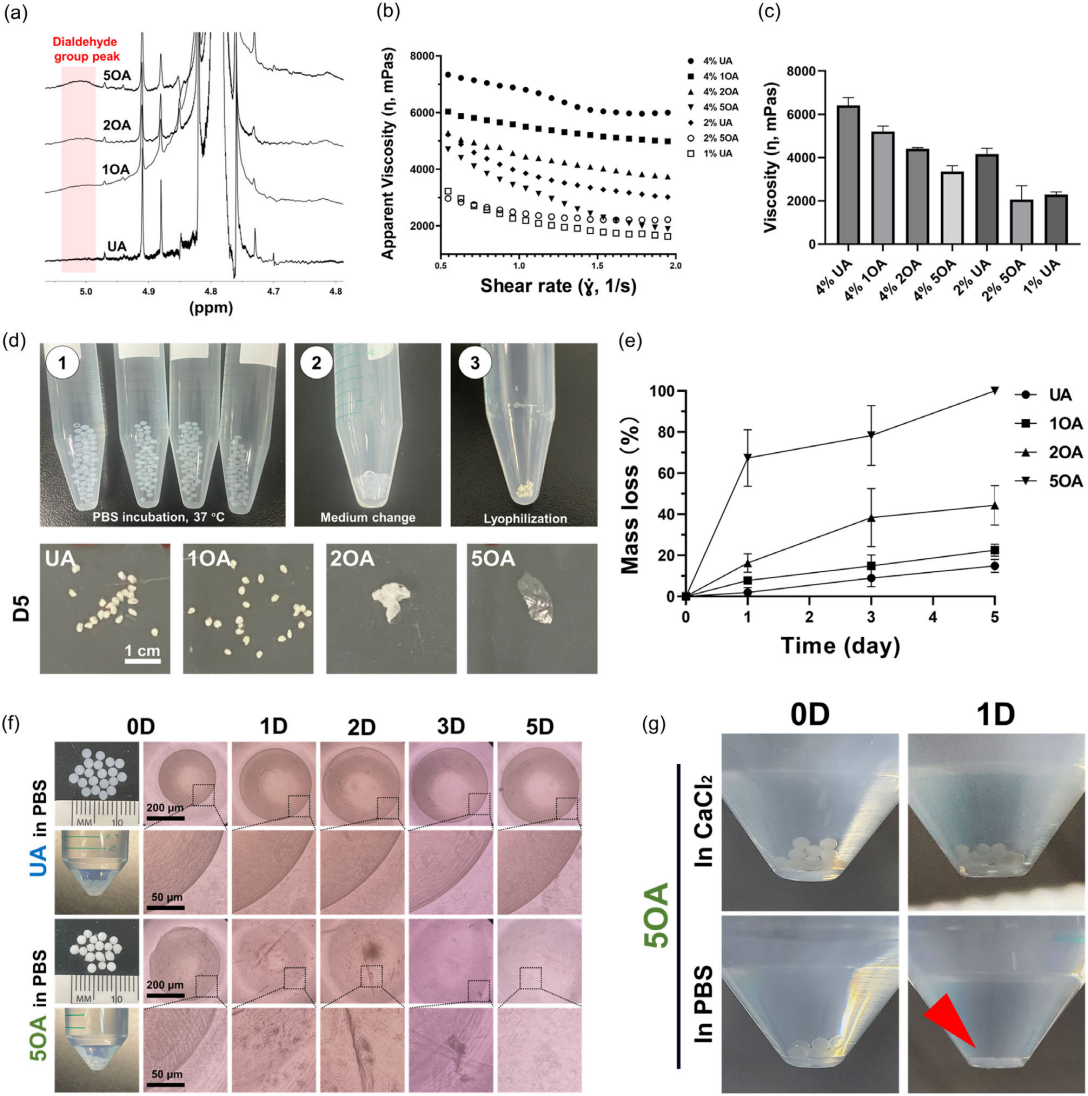
Characterization of OA microgels at different oxidation amounts and concentrations. a) ^1^H NMR spectra confirming oxidation efficiency. A new dialdehyde peak emerged in OA compared to UA, validating the successful oxidation process. b) Steady shear flow curves of OA hydrogel solutions. The dependence of apparent viscosity (*η*) on shear rate ($\dot{\gamma }$) demonstrates shear‐thinning behavior. Network degradation compensates for the concentration effect (conditions: room temperature (25 °C), parallel plate (8 mm), *n* = 3; hydrogel concentrations: 4%, 2%, and 1% w/v in PBS, respectively). c) Effect of concentration and various oxidation on apparent viscosity (*η*) at $\dot{\gamma }$ = 1 s^−1^ for OA microgels (4% UA, 4% 1OA, 4% 2OA, 4% 5OA, 2% UA, 2% 5OA, and 1% UA). Key trends at 4% concentration: UA > 1OA > 2OA > 5OA; UA at different concentrations: 4% > 2% > 1%; 1% UA ≈2% 5OA (the network degradation effect compensates for the concentration effect). d) Degradation test of UA or OA microgels in 15 mL calcium‐free PBS and residue at day 5. Procedures: d1) 40 microgels in a 15 mL conical tube. d2) The medium was changed on a daily basis. d3) Lyophilization followed by weighing. Residual order: UA > 1OA > 2OA > 5OA. e) Degradation kinetics of OA microgels (2% UA, 4% 1OA, 4% 2OA, and 4% 5OA) from days 0 to 5 (*n* = 3). f) Morphological evolution of UA and 5OA microgels (days 0–5). Structural collapse begins at day 1 (OA), with complete degradation by day 5. g) Calcium‐dependent stability: microgels maintained integrity in CaCl_2_ but fully degraded in PBS after 1 day.

The schematic illustration of OA preparation and application is shown in Figure 1a. Unmodified alginate (UA) was oxidized using sodium periodate (NaIO_4_) to alter its chemical structure, enabling self‐degradation.^[^ ^28^ ^]^ Oxidation reactions on the –OH groups at the C‐2 and C‐3 positions of the uronic units of UA resulted in the formation of dialdehyde groups in each oxidized monomeric unit through carbon–carbon bond breaking. This destabilized the polymer backbone, making it susceptible to hydrolytic cleavage in aqueous environments.^[^ ^29^ ^]^ The newly formed dialdehyde groups were confirmed by the ^1^H‐NMR pattern of OA, which revealed a new proton peak around 5 ppm in the spectrum, and it was observed that the peak increased with the increase in oxidation treatment (**Figure** **2**a). This peak was attributed to a hemiacetal proton generated from the hydroxyl groups of the aldehyde and its neighboring groups.^[^ ^30^ ^]^

Next, the viscosity of different concentrations of OA was measured to evaluate injectability. Both UA and 1OA, 2OA, and 5OA exhibited shear‐thinning behavior, allowing for the injection of the macromer solutions prior to cross‐linking. Viscosity measurements revealed the following order: UA > 1OA > 2OA > 5OA (Figure 2b). This result is attributed to polymer chain cleavage upon oxidation, which reduces viscosity and compromises network integrity.^[^ ^31^ ^]^ In Figure 2c, the apparent viscosity showed that a 2% UA solution exhibited a viscosity similar to that of a 4% 2OA solution, while a 1% UA solution was comparable to a 2% 5OA solution. Therefore, OA concentrations were roughly doubled relative to UA concentrations (i.e., 2% OA vs. 1% UA) throughout this study to match the baseline physicochemical behavior of UA.

The degradation profiles of UA and OA microgels were measured. Conditioned microgels fabricated from 2% UA, 4% 1OA, 4% 2OA, and 4% 5OA were monitored over 5 days. Due to the low individual microgel mass, samples comprising ≈40 microgels were collected per time point for wet weight measurement (Figure 2d). In Figure 2e, a gradual degradation trend was observed over time: 5OA > 2OA > 1OA > UA. This phenomenon results from the increased number of dialdehyde groups, which are proportional to the degree of oxidation, thereby accelerating the degradation speed.^[^ ^31^ ^]^ The self‐degradability of OA and UA microgels was characterized over 5 days (Figure 2f). Bright‐field images showed that the OA microgels maintained a spherical shape on day 0. On day 1, the spherical structure began to degrade, with the borders becoming less distinct. On day 2, most of the OA microgel components had disappeared compared to the original structure of the UA microgels. By day 5, the OA microgels were fully gone, while the UA microgels were slightly swollen but retained their original round structure. Similar degradation kinetics were observed in previous studies involving successful oxidation.^[^ ^28^ ^]^ Additionally, the generated OA microgels were randomly selected and placed in CaCl_2_ or Phosphate‐Buffered Saline (PBS) (Figure 2g). On day 1 postincubation, the OA microgels were invisible in PBS, while the original structure was still visible in the CaCl_2_ condition.

The successful modification of alginate‐based materials derived from natural sources presents a promising approach for its use in making tissue‐engineered constructs. Alginate not only provides biodegradability but also alleviates safety concerns associated with synthetic polymers.^[^ ^32^ ^]^ However, UA and other biomaterials often exhibit prolonged degradation times, leading to foreign body reactions, tissue adhesion, impaired tissue regeneration, and even blockage of the circulatory system when used in vivo.^[^ ^33^ ^]^ These complications highlight the importance of developing biodegradable materials that can degrade at an appropriate rate without causing adverse effects. Therefore, our results underscore the potential of OA microgels as a safer and more efficient alternative to synthetic polymers.

### 2.2 Characterization and Optimization of OA Using hMSCs‐TERT

**Figure 3:**
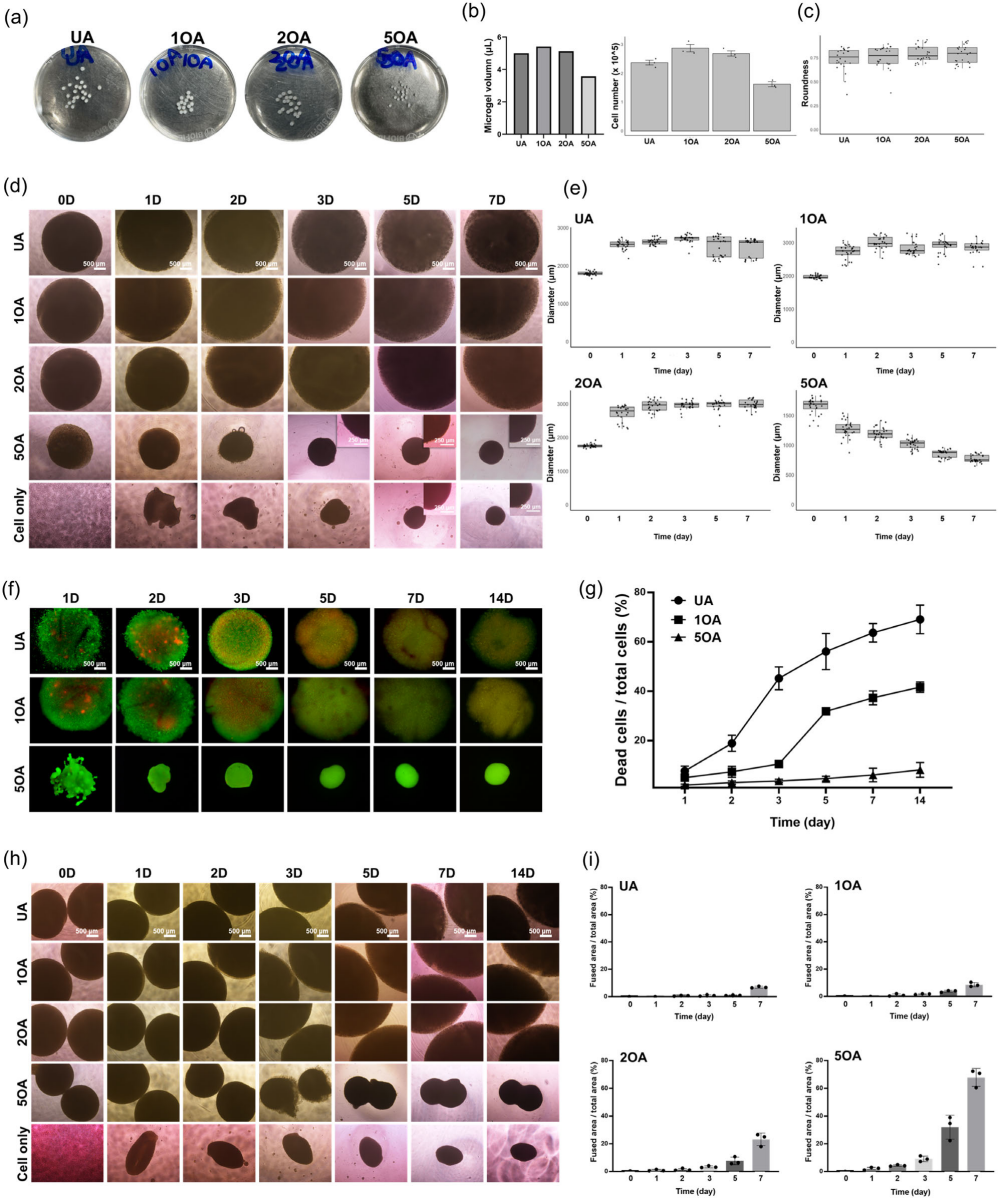
Optimization of OA condition for hMSC‐TERT encapsulation. a) Macroscopic appearance of OA microgels at day 0. b) Count of encapsulated cells in OA microgels (UA, 1OA, 2OA, and 5OA; *n* = 3). c) Quantification of microgel circularity at day 0 (*n* = 24). d) Morphological evolution and e) size distribution of high‐density hMSCs‐TERT encapsulated in microgels (UA, 1OA, 2OA, 5OA, and a seeded cell‐only control) from day 0 to day 14. Key observations: initial swelling (days 0–1) followed by progressive contraction in OA groups; no contraction in UA; no swelling in cell‐only groups (*n* > 25). f) Morphological changes and g) quantification of spontaneous integration behavior of different OA microgels (UA, 1OA, 2OA, 5OA, and a seeded cell‐only control) from day 0 to day 14. Integration capacity: cell‐only > 5OA > 2OA > 1OA > UA. Microgels maintained compartmentalization compared to fused cell‐only controls (*n* = 3). h) Live/dead staining and i) viability quantification of UA, 1OA, and 5OA microgels (days 0–14; *n* = 3). Survival trends: UA and 1OA showed progressive viability loss; 5OA exhibited the highest initial viability with a gradual decline.

To ensure the comparability of UA and OA, we selected 1% UA and 2% concentrations of 1OA, 2OA, and 5OA microgels, which have similar viscosities, to encapsulate human mesenchymal stem cells by human telomerase reverse transcriptase (hMSCs‐TERT). In **Figure** **3**a, UA and OA microgels were cross‐linked to form a single microgel. The size distribution of UA and OA microgels exhibited the following trend: 1OA > 2OA > UA > 5OA. We prepared 200 μL of 1% UA, 2% 1OA, 2% 2OA, and 2% 5OA macromer solution to encapsulate hMSC‐TERT cells at a density of 1.0 × 10^8^ cells mL^−1^ using a dripping–cross‐linking technique for validation of various conditioned OAs. In total, we produced 40, 37, 39, and 56 microgels, corresponding to 5, 5.41, 5.13, and 3.57 μL for the UA, 1OA, 2OA, and 5OA groups, respectively (Figure 3b, left). Meanwhile, the amounts of cells encapsulated in each hydrogel varied partially depending on the volume of each microgel (Figure 3b, right). However, all microgels maintained a spheroidal structure (Figure 3a,c).

To optimize the conditions of OA for stem cell aggregation, we systematically compared the aggregation behavior of hMSCs‐TERT encapsulated in UA and OA microgels, as well as cells seeded in a tissue culture well plate without microgels as a control (Figure 3d). The UA and 1OA groups exhibited an initial swelling phase from days 0 to 1, followed by progressive contraction over the subsequent 7 days. The 2OA group revealed an initial swelling phase from days 1 to 2, after which it continued to swell for 7 days. The UA, 1OA, and 2OA groups showed structural changes, with no decrease in size, indicating that these microgels were not fully degraded. In all microgel groups, the 5OA microgels exhibited a unique decrease in size, aligning with cell condensation kinetics (Figure 3e). This result is attributed to the rapid degradability of 5OA, which facilitated self‐condensation of the cells.^[^ ^34^, ^35^, ^36^ ^]^ The seeding cell‐only group required up to 5 days to form irregular spheroids, but many cells failed to integrate into a 3D cell condensation. Thus, we believe that the confinement provided by 5OA microgels actively guides cells into dense aggregates, minimizing cell loss and creating an environment that promotes survival and maintenance of stemness.^[^ ^37^, ^38^ ^]^ Next, we performed a live/dead test for 14 days using UA, 1OA, and 5OA groups (Figure 3f,g). Nearly all cells in 5OA were clearly viable, with contractions observed from day 1. While UA and 1OA showed a loss of viability, leading to less cell organization over time, this was attributed to the physical advantages of condensed microenvironments that enhance metabolite exchange due to reduced diffusion distances.^[^ ^39^ ^]^ The results from the 5OA group indicated the rapid establishment of cell‐cell contacts, creating a favorable 3D cell‐only environment.

In further investigation, we hypothesized that the self‐degradability of 5OA facilitated tissue integration. In the UA and OA microgel groups, different integration capacities were observed (Figure 3h,i). When contracted, only the 5OA group exhibited spontaneous 3D tissue integration, forming integrated structures that retained the compartmentalization at the interface and preserved the spatial organization of individual condensates. Whereas 3D cell condensations were also formed in cell‐only controls, their formation was delayed and the cell masses appeared disorganized and homogenous. The fusion capability of 5OA microgels holds significant implications for recapitulating tissue–tissue interactions, such as epithelial–mesenchymal interactions (EMIs), a fundamental requirement for the formation of various organs including the tooth.^[^ ^40^ ^]^ Based on the results, we established that 5OA is the optimal condition for generating 3D dental stem cell aggregates, exhibiting the fastest degradation rate and improved spontaneous aggregation behavior.

### 2.3 Formation of SCAP Aggregates by Single and Double UA/SCAP and 5OA/SCAP Microgels

**Figure 4:**
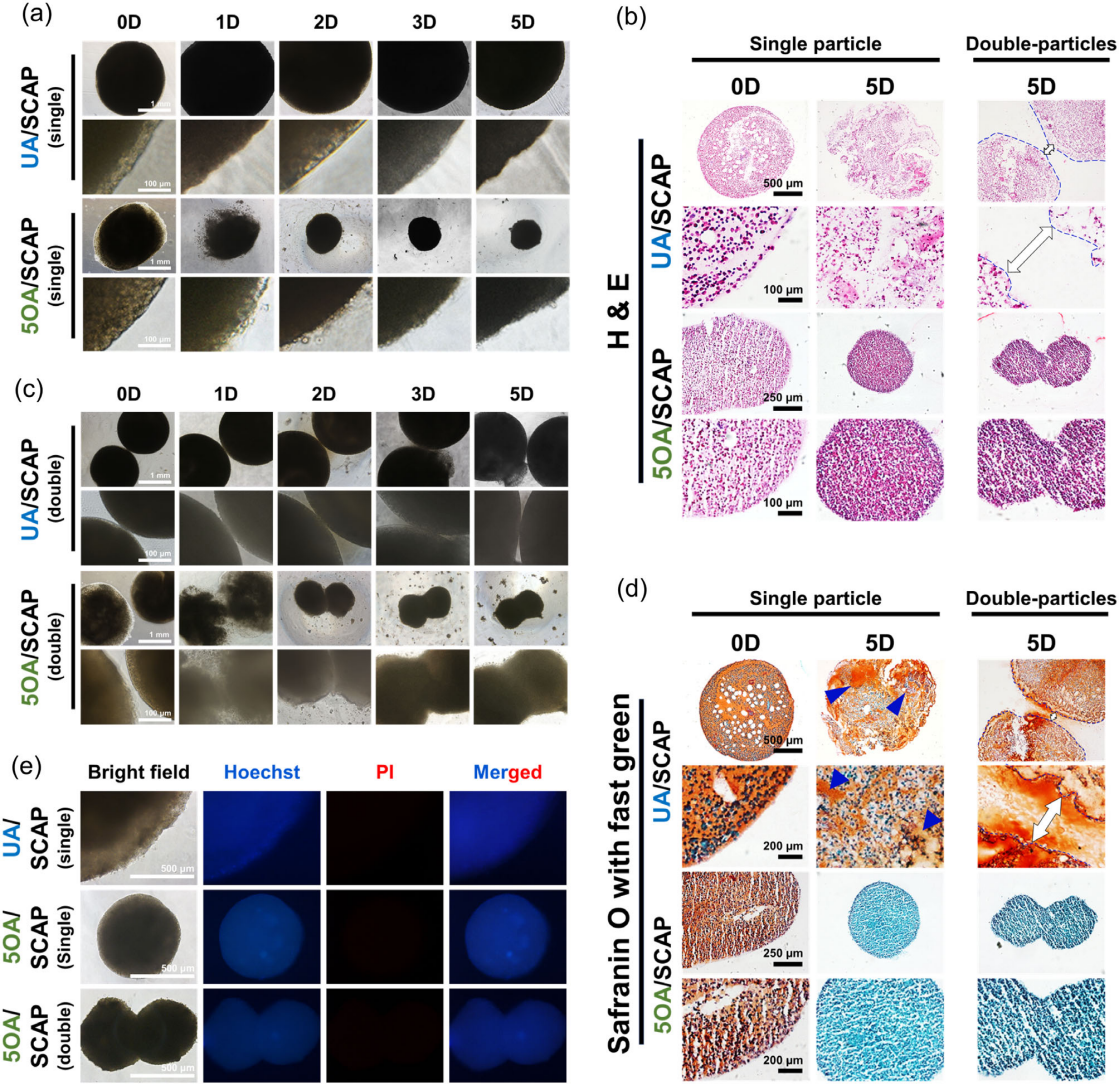
Microscopic observation of the self‐condensation process of SCAP aggregates in OA from day 0 to day 5. a) Self‐assembly began on day 1 through the degradation of 5OA, while UA did not degrade and did not form aggregates. b) H&E staining of 5OA/SCAP aggregates at day 0 and day 5 for both single and double spheroids. Blue dashed lines indicate the borders of high‐density SCAP‐laden UA (UA/SCAP), while white arrows denote the gaps between UA/SCAP. No gaps were observed between 5OA/SCAP aggregates. c) Microscopic observation of SCAP aggregate fusion between double 5OA/SCAP from day 0 to day 5. The UA/SCAP did not facilitate aggregate fusion. d) Safranin O with fast green staining revealed residual alginate components in the UA/SCAP group as a strong red color (blue arrows). Individual SCAPs were embedded in UA and 5OA on day 0. However, no residual alginate was observed in the 5OA/SCAP group at day 5. Blue dashed lines indicate the borders of UA/SCAP, while white arrows denote the gaps between UA/SCAP. e) Hoechst/PI staining of UA/SCAP and 5OA/SCAP. Living cell fusion was clearly observed in the 5OA/SCAP group.

Using conventional microgel generation methods, SCAPs were successfully encapsulated and evenly distributed within the UA or 5OA matrix on day 0, demonstrating the potential of this microgel for high‐density cell delivery (**Figure** **4**a). Our characterization of the microgel using hMSCs‐TERT revealed that the volume ratio of UA to OA is ≈1.4 (Figure 3b). To ensure comparability cell number within microgel between two groups, the concentration of cells for 5OA was roughly increased to 1.5 times as that of UA. On day 1, the transparent border of the 5OA microgel became less distinct but maintained a rough structure, with the spheroid shrinking to indicate the beginning of condensation. In contrast, the UA aggregate showed swelling of the spheroid, with the border remaining clear throughout the culture period. By day 2, 5OA aggregates began to form, indicating cell condensation with less transparent hydrogel components. After 5 days of culture, the 5OA microgels were fully degraded, leading to the formation of high‐density SCAP cell condensation. Over time, the morphology of the condensed cells transformed into a spherical shape. In contrast, the structure of the UA microgels remained unchanged throughout the observation period, indicating swelling rather than cell condensation.

In Figure 4b, H&E staining demonstrated clear condensation of SCAP into aggregates, with cells tightly packed on day 5. In contrast, the cells in the UA group remained loosely distributed, with no evident cell–cell communication observed between days 0 and 5. Safranin O with fast green staining revealed no residual material in the high‐density SCAP‐laden 5OA (5OA/SCAP) group (Figure 4d and S1, Supporting Information), as no carboxyl groups were detected in orange by Safranin O,^[^ ^41^ ^]^ providing a clear environment for cell growth and interaction. However, in the UA group, carboxyl groups in residual alginate were prominently stained a strong orange color (blue arrows), which interfere with cell–cell interaction and disrupt tissue organization.^[^ ^42^ ^]^

We also observed the spontaneous integration of two 5OA/SCAP microgels over a 5‐day period (Figure 4c,d). On day 0, the double microgels were in contact but had distinct boundaries. By day 1, the SCAP from the OA microgels began to integrate, and the border between the two 5OA/SCAP aggregates became blurred as the outer shell of the OA microgel started to break down, indicating partial fusion of the two structures. The microgels continued to merge over the following days, culminating on day 5. Additionally, H&E staining clearly confirmed the integration of single aggregates into assembloids, with no residual alginate components observed in the Safranin O with fast green staining. In contrast, in the UA group, residual alginate prevented the spheroids from fusing together (Figure 4b–d).

The 5OA‐based microgel system facilitates rapid formation of 3D spherical clusters. This process is driven by cell affinity to each other and the forces within their structures.^[^ ^43^ ^]^ Upon initial contact, cells undergo cytoskeletal rearrangement, which enhances adhesion and promotes tighter connections.^[^ ^44^ ^]^ The changes in shape of the 5OA/SCAP microgel demonstrate its ability to encourage natural cell aggregation, creating artificial tissues that closely resemble their natural counterparts.^[^ ^43^ ^]^ Moreover, the double spheroid method can be used to model tissue interactions during organ growth and disease and to engineer complex tissues with multiple components.

To examine the viability of condensed SCAP released from the 5OA microgels, Hoechst/PI analysis was performed on day 5 (Figure 4e). The results showed that most SCAPs were viable, indicating excellent biocompatibility of both the 5OA and UA systems. This outcome contrasts with that observed for hMSC‐TERT (Figure 3f,g), a difference that may be attributed to SCAP's high inherent cell viability and natural adaptability to hypoxic conditions,^[^ ^45^ ^]^ which allow them to continue to proliferate under oxygen deprivation. A similar result was observed in the integrated SCAP assembloids, demonstrating that the integration of spheroids was accompanied by high cell viability, which is crucial for stem cell‐based 3D tissue transplantation.^[^ ^46^ ^]^ Cell‐based therapies operate through multiple mechanisms, such as cellular differentiation, secretion of bioactive molecules like growth factors and cytokines, regulation of immune responses, and hastening tissue repair and remodeling.^[^ ^47^ ^]^ Thus, it is of paramount importance to secure a sufficient amount of high‐quality stem cells with good stemness, viability, and proliferation rates. These results suggest that this hydrogel system holds potential for the fabrication of aggregates and could be employed to induce condensation, facilitating the in vitro and in vivo formation of dental tissue‐like structures.

### 2.4 Transplantation of UA/PDLSC and 5OA/PDLSC Microgels in Mice Subcutaneously

Building on the potential for in vitro self‐aggregation, we further investigated the self‐tissue integration process of hDSC‐laden 5OA microgels in the bodies of mice. For comparison, we prepared precultured high‐density PDLSC‐laden UA (UA/PDLSC) and high‐density PDLSC‐laden 5OA (5OA/PDLSC). The PDLSC‐laden UA (UA/PDLSC) and OA microgels (5OA/PDLSC) were generated and immediately implanted subcutaneously in mice alongside the precultured UA/PDLSC and 5OA/PDLSC from 2 days prior (**Figure** **5**a). After 2 days of culture, we observed swollen precultured UA/PDLSC and self‐condensed precultured 5OA/PDLSC (Figure 5b). Surgery was carefully performed for a four‐quadrant subcutaneous transplantation (Figure 5c). Two days post‐transplantation, both UA/PDLSC groups exhibited swelling, while both 5OA/PDLSC groups maintained normal skin volume (Figure 5d). The transplants and surrounding tissue were then harvested. Examination of the harvested tissue revealed that, in the UA groups, the UA microgels retained their structure without significant degradation (Figure 5e). This was consistent in both the precultured and immediately transplanted sites, confirming previous finding that the UA microgels were nondegradable and remained intact.^[^ ^33^ ^]^ The UA/PDLSC microgels were easily removed from the body and could be clearly identified under bright‐field microscopy. In contrast, despite the brief 2 day implantation period, both OA groups achieved complete tissue fusion, making the shape of any residual OA microgels indiscernible. This outcome suggests that the OA microgels were fully degraded, allowing the transplanted cells to integrate effectively with the surrounding tissues.^[^ ^30^ ^]^ In Figure 5f, high‐magnification microscope images revealed the presence of blood vessels at the tissue and implant sites across all time points in OA groups, indicating integration of the transplanted cells with host tissues as the 5OA degraded. In contrast, in the UA groups, although some residual UA microgels showed gradual degradation, they remained encapsulated by the surrounding tissues and were detectable for a longer duration. Furthermore, human‐specific nuclear antigen staining confirmed the human origin of the subcutaneously implanted graft (Figure S2, Supporting Information). This result underscores the reliability of the xenogeneic mouse models, providing a solid foundation for subsequent experimental analyses.

Figure 5Transplantation of precultured or immediately prepared UA and 5OA encapsulated PDLSC microgels (UA/PDLSC versus 5OA/PDLSC) subcutaneously in mice. a) Schematic illustration of the transplantation surgery and grouping. b) Microscopic image showing UA/PDLSC microgels and PDLSC aggregates from 5OA after 2 days of in vitro preculture. c) Transplantation of each group in the four quadrants of the subcutaneous area. 1) Administration of anesthesia and analgesics. 2) Surgical site hair removal. 3) Four‐quadrant transplantation. 4) Graft placement in the subcutaneous pocket. 5) Wound closure. 6) Postoperative care. Gross images of the transplanted microgels before tissue dissection show the structure of the precultured UA and 5OA compared to the instantly cross‐linked and transplanted UA and 5OA. d) Samples collected on day 2 showed complete degradation of the hydrogel and solid 3D tissue formation. e) Optical gross images and f) stereomicroscopic images of the collected samples. The UA in both groups remained in place without integrating with the surrounding tissues, while all 5OA groups (precultured 5OA/PDLSC and 5OA/PDLSC) demonstrated complete integration and bonding between the transplant and host tissues.

**Figure:**
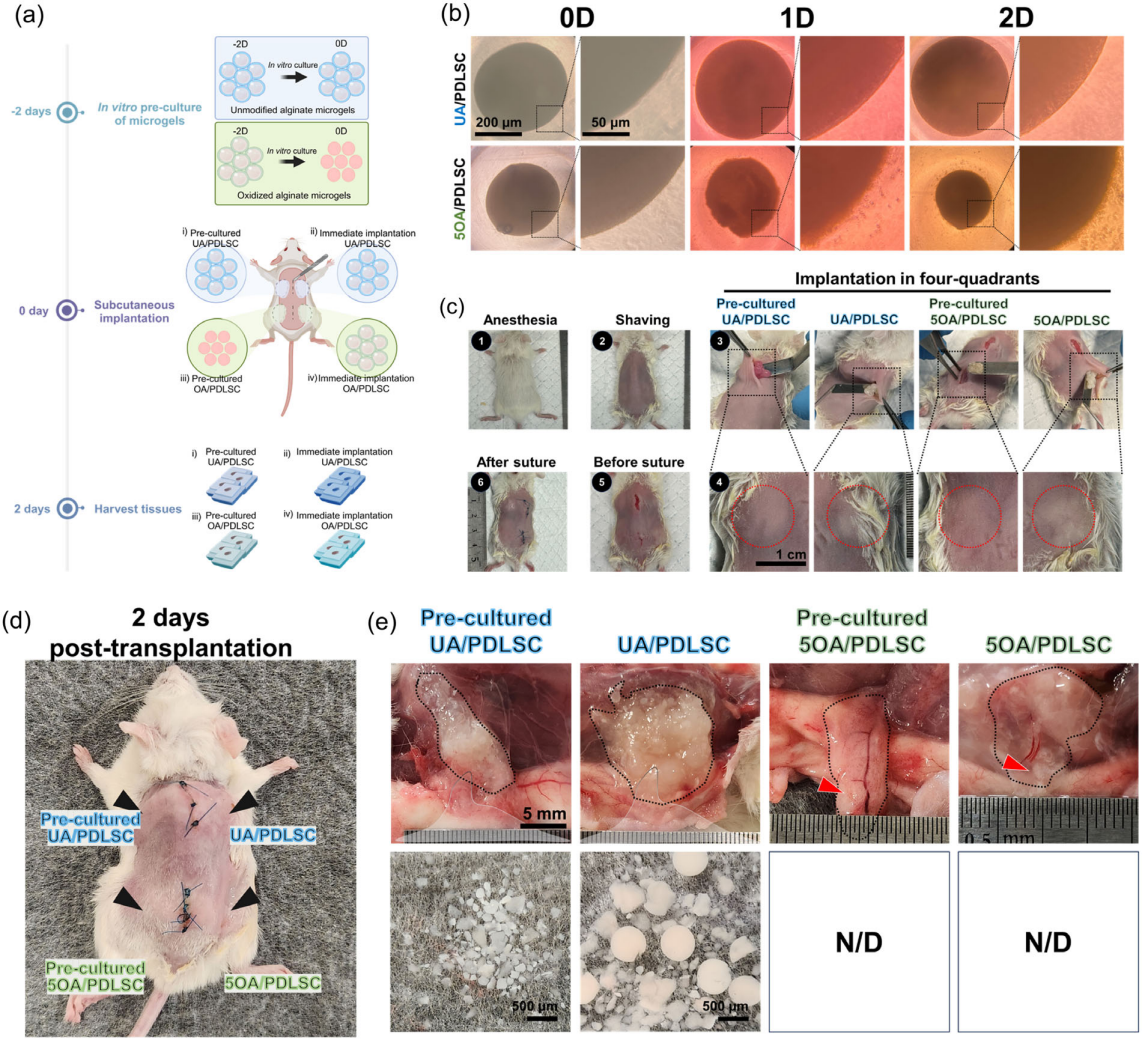

**Figure:**
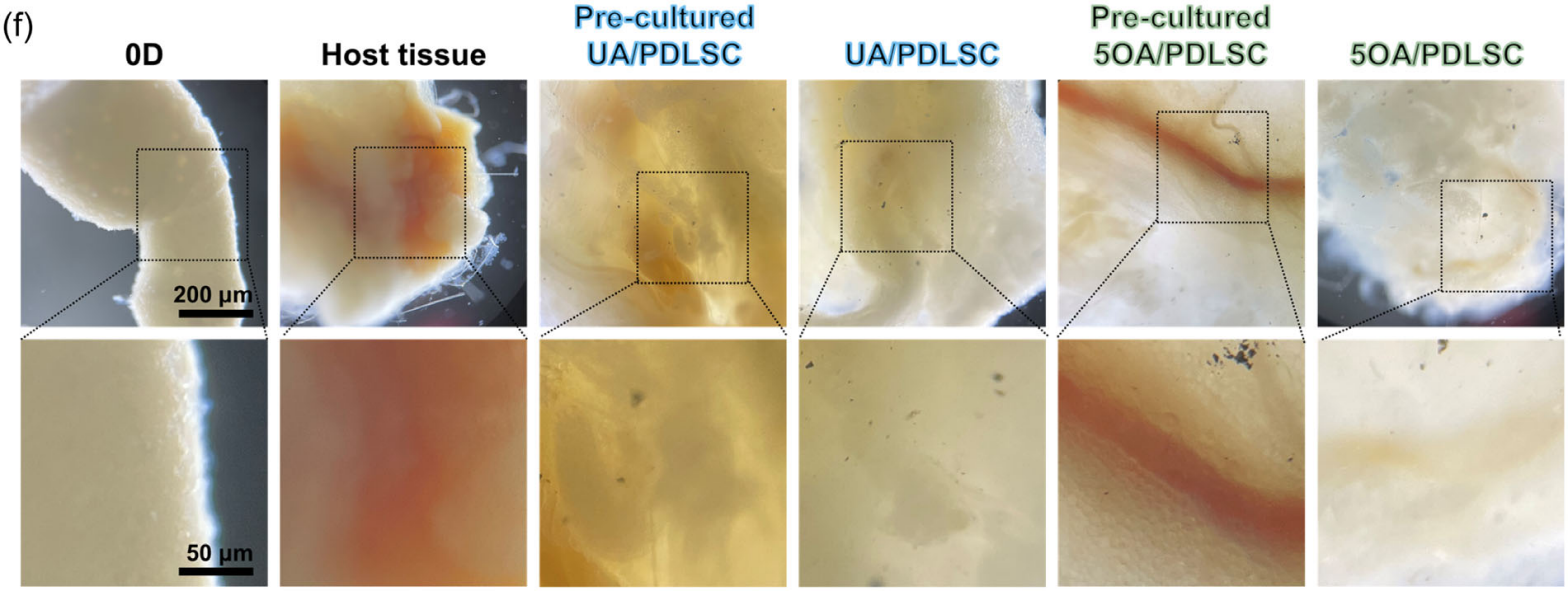

**Figure 6:**
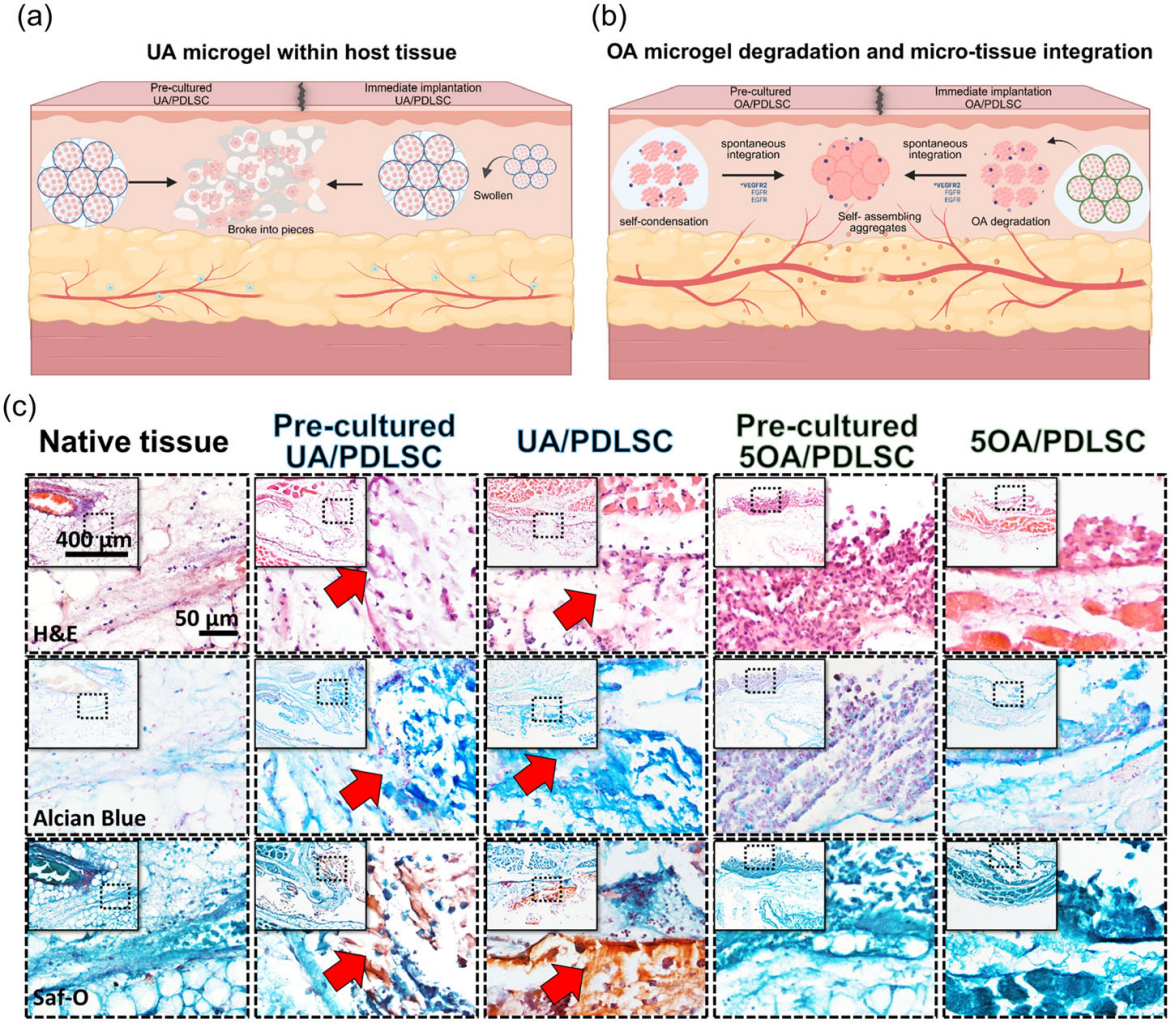
In vivo integration of precultured and immediate implantation of UA/PDLSC 5OA/PDLSC microgels. a,b) Schematic illustrations depict the grouping, self‐condensation, and spontaneous integration processes of (a) UA and (b) 5OA under in vivo conditions. c) Histological analysis conducted 2 days postimplantation using H&E, Alcian blue/nuclear fast red, and Safranin O/fast green staining of host tissue and all transplanted groups, including precultured UA/PDLSC, immediately implanted UA/PDLSC, precultured 5OA/PDLSC, and immediately implanted 5OA/PDLSC. In the UA groups, residual microgel fragments (indicated by red arrow) disrupted the integration of native tissue. In the OA groups, complete degradation of microgels was observed, resulting in seamless interfaces between the graft and native tissue.

Next, the collected samples were histologically analyzed (**Figure** **6**). Figure 6a,b presents schematic diagrams illustrating the proposed mechanism for the transformation of UA and 5OA microgels after subcutaneous transplantation in mice, respectively. Abundant residual alginate in both precultured and direct transplantation UA groups was detected, as shown by the strong red staining from Safranin O (Figure 6c). Residual UA trapped individual PDLSCs and prevented them from integrating with host tissues. In contrast, OA groups (precultured or immediately transplanted 5OA/PDLSC) exhibited the formation of PDLSC condensations integrated with mouse connective and muscle tissues. Notably, the precultured 5OA/PDLSC presented a more robust condensation, likely due to prior in vitro organization into 3D tissue. Nevertheless, the immediately transplanted 5OA/PDLSC group also displayed clear signs of condensation, suggesting that the high‐density PDLSC aggregates formed spontaneously as the 5OA degraded. These results demonstrated that the 5OA microgels can be fully degraded within 2 days post‐transplantation, presumably through similar processes as observed in vitro, which allows the timely integration of embedded cells with the host tissue. Although numerous studies have confirmed the successful degradation of OA, some cases report remnants of the hydrogel persisting due to differences in nutrient exchange between in vivo and in vitro conditions.^[^ ^48^ ^]^ In this study, we observed neither signs of graft failure nor remaining material.

### 2.5 In Vivo Vascularization and Tissue Organogenesis of 5OA/PDLSC Microgels for 3 weeks

**Figure 7:**
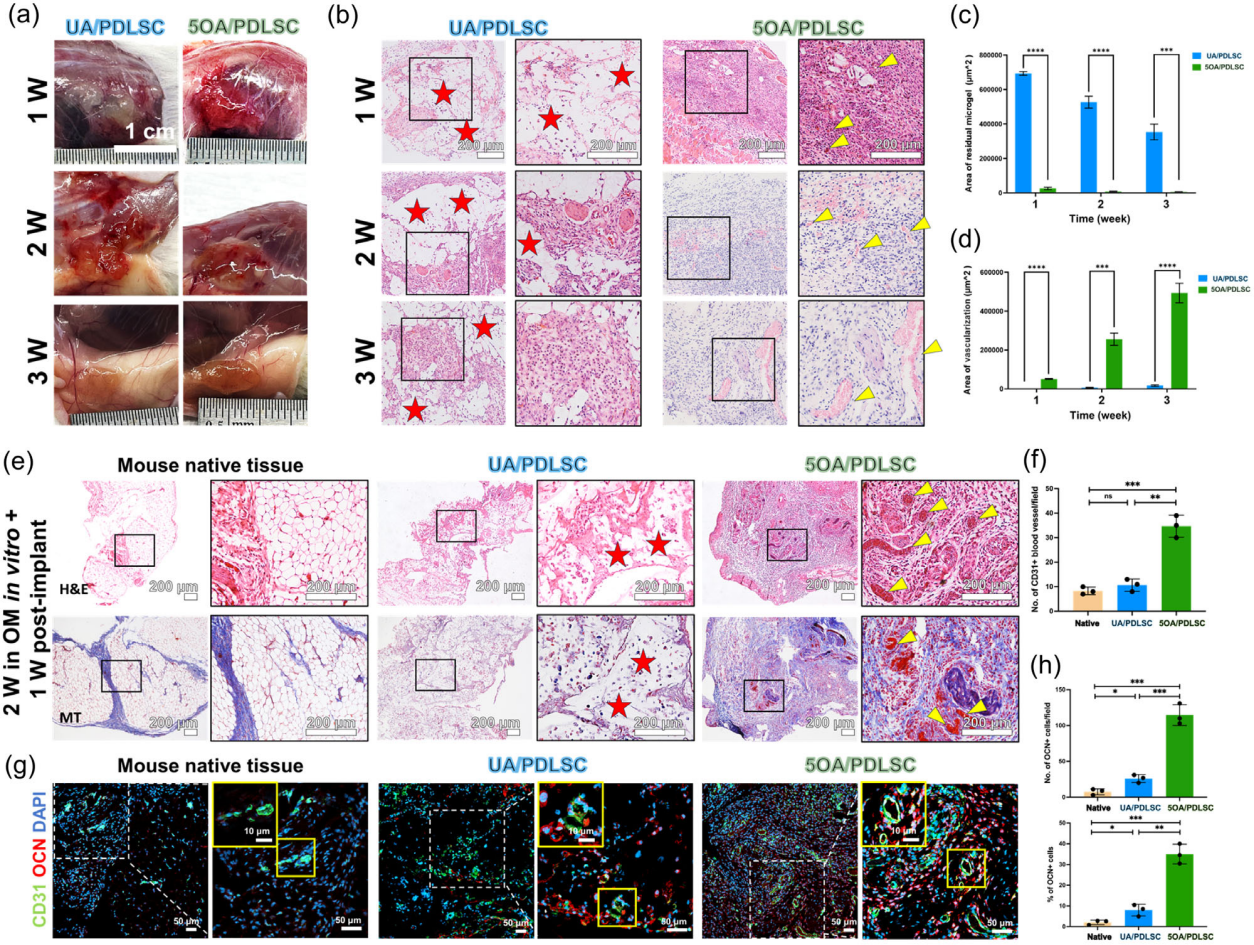
Transplantation of UA/PDLSC and 5OA/PDLSC demonstrated tissue integration and vascularization. a) Gross images and b) H&E staining confirmed tissue integration and vascularization. c) The area of residual microgel and ingrowth of vascular structures were quantified based on H&E staining. The UA group showed gradual degradation, with 50% residual material at 3 weeks, while the 5OA group had no detectable residue. d) Quantification of vasculature in the 5OA/PDLSC group was compared with the UA/PDLSC group (*n* = 3). e) H&E and Masson's trichrome staining were performed 1 week post‐transplantation of 2 week osteogenic precultured 5OA/PDLSC and UA/PDLSC. f) Quantification of CD31‐positive blood vessels per field indicated ≈4.2‐fold and 3.3‐fold increases in vascularization in the 5OA group compared to the native and UA groups, respectively (*n* = 3). g) Immunofluorescent staining of CD31 (green) and OCN (red) demonstrated vascularization and osteogenic differentiation of PDLSC. h) Quantification of OCN‐positive cells and their percentage per transplant field revealed that the 5OA group had ≈15.0‐fold and 4.4‐fold more OCN‐positive cells than the native and UA groups. A similar trend was observed for the percentage of OCN‐positive cells, with increases of ≈16.6‐fold and 4.4‐fold (*n* = 3). OCN: Osteocalcin.

To evaluate the translational potential, freshly prepared 5OA microgels encapsulating human PDLSCs were directly transplanted and analyzed at 1, 2, and 3 weeks. Gross images and H&E staining revealed rapid host microvessel infiltration (yellow arrow) into the 5OA microgels (**Figure** **7**a,b). However, the UA/PDLSC group exhibited numerous acellular spaces, indicating the presence of undegraded UA (red star). Over time, the amount of residual UA appeared to decrease due to fragmentation, but it remained in site after 3 weeks (Figure 7c). Additionally, the quantity of infiltrating blood vessels was greater in the 5OA group compared to the UA group (Figure 7d). This phenomenon is attributed to the incomplete degradation of UA, which impeded tissue infiltration from surrounding tissues to the implant. This finding is crucial for reconstructing vascularized tissue, as a supply of blood vessels can enhance tissue viability and promote differentiation through the delivery of oxygen, nutrients, and growth factors.^[^ ^49^ ^]^ For example, studies have demonstrated the promotion of angiogenesis through the implantation of alginate microgels and the passive acceleration of alginate degradation using chelating agents.^[^ ^42^ ^]^ In contrast, our approach utilized self‐degradable 5OA microgels laden with high‐density PDLSCs to promote cellular condensation‐driven tissue reorganization and facilitate blood vessel infiltration.

We further evaluated whether the organized 3D tissue maintained stemness and retained osteogenic potential using a combination of 2 week osteogenic culture in vitro and 1 week subcutaneous implantation. Transplanted 5OA/PDLSC microgels exhibited robust collagen deposition and blood vessel infiltration (yellow arrow in Figure 7e). Quantification of CD31‐positive vessel density confirmed a 4.2‐fold and 3.3‐fold increase in vascularization in the 5OA group compared to the native host and UA groups, respectively (Figure 7f; *p* < 0.0001). This finding can be explained by the rapid degradation of 5OA, which is expected to release PDLSC‐secreted angiogenic factors (e.g., VEGF/Ang‐1) that recruit host vasculature, a mechanism well supported by the literature.^[^ ^50^, ^51^, ^52^ ^]^

In the immunofluorescence staining (Figure 7g), the 5OA group demonstrated clearly vascularized lumen structures and a significant presence of osteocalcin (OCN)‐positive cells. In contrast, while OCN‐positive cells were also observed in the UA group, these cells appeared poorly formed and disorganized. This disorganization is believed to be due to the residual hydrogel, which corresponds to the earlier findings. Immunofluorescence quantification revealed a 15.0‐fold and 4.4‐fold increase in OCN‐positive cells in the 5OA group compared to the native and UA groups, respectively, with the proportion of OCN‐positive cells increasing 16.6‐fold and 4.4‐fold (Figure 7h; *p* < 0.001). Therefore, the 5OA encapsulating cell system uniquely facilitates simultaneous degradation, vascularization, and differentiation, achieving functional osteogenesis and enhancing the potency of precultured grafts. Its “degrade‐to‐activate” paradigm offers transformative potential for minimally invasive dental tissue regeneration.

### 2.6 Construction of EPI + MES Assembloid Tooth Germ Model via 5OA Microgels

**Figure 8:**
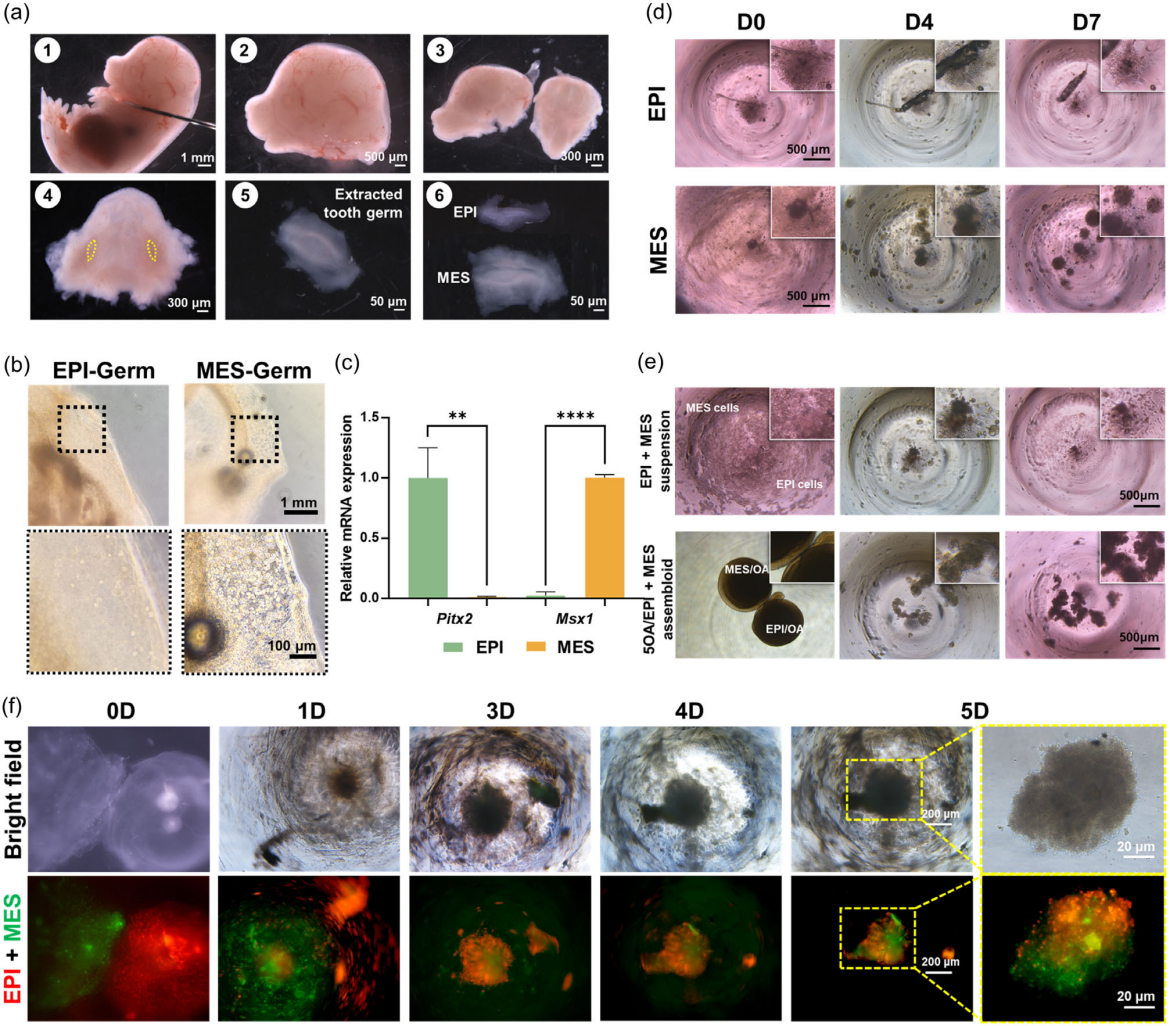
Generation of bioengineered tooth germ model using 5OA microgels. a) Epithelial and mesenchymal tooth germs were dissected from the lower jaw of E14.5 mouse embryos. 1) Transfer the mouse embryo into a Petri dish with cold PBS and use needles to separate the head from the body. 2) Transfer the isolated head to a glass Petri dish with cold PBS. 3) Separate the upper and lower jaws using needles. 4) Remove the tongue and cervical vertebrae from the lower jaw to visualize the molar tooth germs. 5) Dissect the developing tooth germs and transfer them to a smaller glass Petri dish with cold PBS. 6) Use dispase II to remove the basement membrane and separate the dental epithelium from the dental mesenchyme. b) Epithelial and mesenchymal tooth germs were digested into single‐cell suspensions and encapsulated within 5OA microgels to form EPI‐Germ and MES‐Germ at day 0. c) Expression levels of the epithelial marker *Pitx2* and the mesenchymal marker *Msx1* were validated by RT‐qPCR in epithelial and mesenchymal cells at day 0. Error bars indicate standard deviation from two independent experiments, each performed in triplicate. d) Morphological analysis revealed a strong self‐organization capacity of mesenchymal cells, whereas epithelial cells produced only smaller and loosely aggregated pellets after 7 days of in vitro culture. e) Morphological analysis showed stronger cell condensation in the 5OA/EPI + MES assembloid group compared with the EPI + MES cell suspension group. f) Bright‐field and fluorescence images visualized the fusion of DiI‐labeled EPI‐Germ and DiO‐labeled MES‐Germ during 5 days of in vitro culture.

To create a bioengineered tooth germ model, epithelial (EPI) and mesenchymal (MES) tooth germs were dissected from the lower jaw of embryonic day 14.5 mouse embryos (**Figure** **8**a). Epithelium and mesenchyme of the tooth germs were separated and then digested into single‐cell suspensions and encapsulated within 5OA microgels to form EPI spheroids (EPI‐Germ) and MES spheroids (MES‐Germ) (Figure 8b). To validate the identity of the isolated dental epithelial and mesenchymal cell populations, mRNA expression of the dental epithelial marker *Pitx2* and the dental mesenchymal marker *Msx1* was analyzed by reverse transcription quantitative polymerase chain reaction (RT‐qPCR) (Figure 8c). *Pitx2* is a transcription factor strongly expressed in the dental epithelium from the bud stage onward, where it plays critical roles in establishing dental epithelial identity, regulating cell proliferation, and driving epithelial invagination.^[^ ^53^, ^54^, ^55^ ^]^ In contrast, *Msx1* is specifically expressed in the dental mesenchyme and is essential for mesenchymal condensation, EMI signaling, and tooth germ morphogenesis.^[^ ^53^, ^56^, ^57^ ^]^ RT‐qPCR analysis confirmed the enrichment of *Pitx2* in the epithelial cell population and *Msx1* in the mesenchymal cell population, thereby verifying the identities of the isolated dental epithelial and mesenchymal cells.

When epithelial and mesenchymal cells were cultured in vitro without 5OA microgels, isolated mesenchymal cells exhibited self‐organization capacity, forming compact condensations after 7 days of in vitro culture, whereas epithelial cells produced only smaller and loosely aggregated pellets (Figure 8d). The interaction between the dental epithelium and mesenchyme is crucial for the progression of tooth development. To evaluate the EPI + MES reconstitution potential using the microgel system, embryonic epithelial cell‐laden 5OA microgels (5OA/EPI) and embryonic mesenchymal cell‐laden 5OA microgels (5OA/MES) were cocultured in V‐shaped 96‐well plates to form 5OA/EPI + MES assembloids (Figure 8e). Morphological analysis revealed that the 5OA/EPI + MES assembloid group exhibited pronounced cell condensation compared with the EPI + MES cell suspension group after 7 days of in vitro culture. To visualize the fusion process, DiI‐labeled EPI‐Germ and DiO‐labeled MES‐Germ were cocultured in V‐shaped 96‐well plates (Figure 8f). Bright‐field and fluorescence imaging showed that the boundary between EPI‐Germ and MES‐Germ became indistinct by day 1 and exhibited initial fusion by day 3 and extensive fusion by day 5 in vitro. These results demonstrated that 5OA microgels are effective in facilitating the condensation of embryonic dental stem cells and EMI, both of which are crucial for embryonic odontogenesis.

Tooth loss is traditionally managed with dentures, bridges, or dental implants. While advancements in new materials and manufacturing procedures have greatly improved the techniques for creating these dental appliances over the past few decades, they still cannot fully replace the functions of a natural tooth, such as sensation.^[^ ^58^, ^59^ ^]^ Whole tooth regeneration through bioengineering methods has the potential to achieve the goal of regaining all the functions of a lost tooth. One promising approach for tooth regeneration harnesses the remarkable ability of embryonic dental cells to self‐organize.^[^ ^60^, ^61^ ^]^ Even after being dissociated into single cells, the cells from the tooth germ can reaggregate, self‐organize, and continue their developmental program to form tooth structures. Ikeda et al. transplanted a bioengineered tooth germ composed of embryonic dental MES and dental EPI within a collagen gel scaffold into the socket of an extracted maxillary first molar.^[^ ^62^ ^]^ The bioengineered tooth germ not only developed into a full tooth with a crown and root, but was also erupted and established occlusion with the opposing lower molars. However, the recombination of reaggregated dental EPI and MES cells is technically challenging due to the lack of mechanical strength in these cell aggregates.^[^ ^63^, ^64^ ^]^ Moreover, the use of a collagen matrix, which is an animal product, poses potential risks such as foreign body reactions.^[^ ^65^, ^66^ ^]^ We aim to address this issue with 5OA by providing mechanical support through the embedding of cell aggregates while facilitating the crosstalk between EPI‐Germ and MES‐Germ, which is necessary for the progression of the odontogenesis program.

To characterize the structure and identity of the EPI + MES assembloids, we first examined their histological features by H&E staining (**Figure** **9**a). The results revealed densely condensed MES cells and loosely arranged EPI cells, with a translucent central region at the site of overlap. We next analyzed the signature gene expression patterns within the EPI + MES assembloids. In situ hybridization for *Pax9* confirmed the preserved dental MES identity within the MES compartment (Figure 9b). E‐Cadherin, an adhesion molecule abundantly expressed in EPI tissues, was detected by immunofluorescent staining, verifying the retained EPI characteristics in the EPI compartment (Figure 9c). Specifically, E‐cadherin fluorescence intensity along a line spanning both EPI and MES regions revealed high expression restricted to the EPI compartment (Figure 9d), indicating minimal intermixing between cells from each compartment. We then performed RT‐qPCR to assess the mRNA expression levels of *Pitx2* and *Msx1* over time in the EPI + MES assembloids (Figure 9e). Compared to the EPI + MES cell suspension group, the 5OA/EPI + MES assembloid group exhibited significantly higher *Pitx2* expression on day 2, as well as elevated *Msx1* expression on both day 2 and day 7. These results suggest that 5OA microgels promote the establishment and maintenance of tooth germ identity in vitro. Overall, these findings support that our 5OA‐encapsulated tooth germ system has strong self‐organization capacity and well‐preserved EPI and MES identities after in vitro culture, highlighting its potential as a tooth germ model for future tooth regeneration applications.

Figure 9In vitro EPI + MES assembloids and in vivo transplantation under the mouse renal capsule. a) H&E staining showed condensed EPI and MES cells, with a translucent area at the center of the overlapping region. b) Frozen sections of EPI + MES assembloids were hybridized with a *Pax9* antisense RNA probe, detected with NBT/BCIP (purple), and counterstained with fast red. c) Immunofluorescence revealed strong E‐cadherin expression within the EPI regions of EPI + MES assembloids. Dashed boxes indicate areas shown at higher magnification. d) E‐Cadherin fluorescence intensity was quantified along a line spanning both EPI and MES areas. e) *Pitx2* and *Msx1* mRNA expression in EPI + MES cell suspension and 5OA/EPI + MES assembloid groups was assessed by RT‐qPCR on days 0, 2, 4, and 7. f) Schematic of the mouse renal capsule transplantation. Embryonic tooth germ, EPI + MES cell suspension, and 5OA/EPI + MES assembloids were collected after overnight culture and transplanted under the renal capsule of NSG mice for 2 weeks before histological analysis. g) Bright‐field imaging, H&E staining, and Masson's trichrome staining of embryonic tooth germ, EPI + MES cell suspension, and 5OA/EPI + MES assembloid groups revealed the histological morphology of the newly formed tissues. h) Immunofluorescence staining of CD31 and OCN in cryosections revealed the formation of blood vessels and bone‐like structures in the 5OA/EPI + MES assembloid group.

**Figure:**
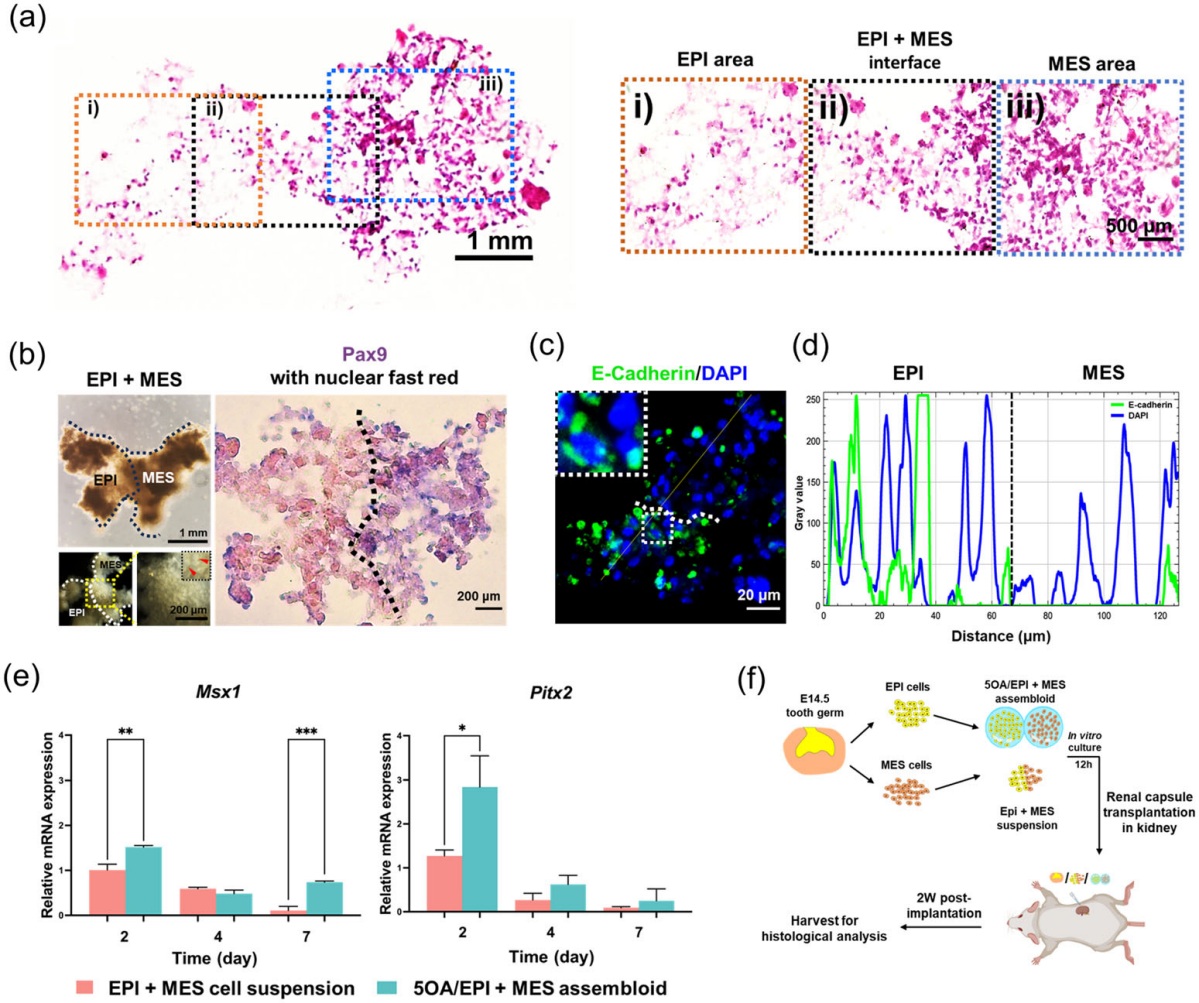

**Figure:**
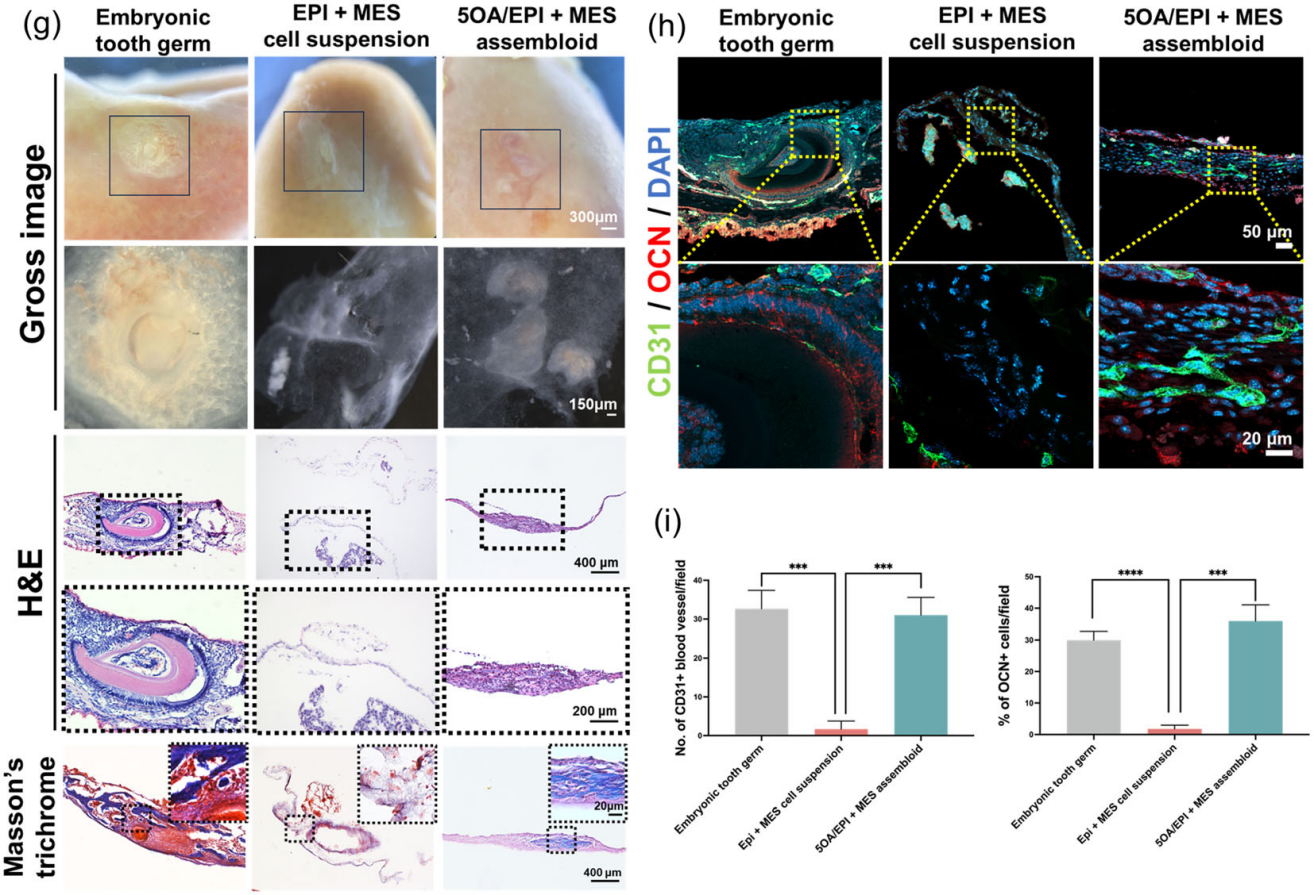

### 2.7 Evaluation of Reconstituted EPI–MES Tooth Germs via In Vivo Transplantation Using 5OA Microgels

To investigate the in vivo functionality of reconstituted EPI + MES tooth germs, EPI and MES cells encapsulated in OA microgels were cocultured overnight in vitro and subsequently transplanted under the mouse renal capsule for 2 weeks (Figure 9f). Whole embryonic tooth germs and EPI + MES cell suspension were also cultured overnight and transplanted under the renal capsule as positive and negative controls, respectively. To characterize the structural features of the newly formed tissues, stereomicroscopy and histological analyses using H&E and Masson's trichrome staining were performed (Figure 9g). After 2 weeks of transplantation, embryonic tooth germs generated well‐organized tooth structures in vivo, comprising multiple dental tissues, including dental pulp, dentin, enamel, periodontal ligament, and blood vessels. The 5OA/EPI + MES assembloid group gave rise to bone‐like tissues, in which histological examination revealed mineralized matrices, newly deposited collagen fibers, and trabecular architecture. The EPI + MES cell suspension group, however, formed indistinguishable loose connective tissues without little structure. To further assess the identity of the newly formed tissues, markers of vascularization and osteogenesis were examined by immunostaining (Figure 9h). These analyses confirmed that both the dentin‐like structures formed by embryonic tooth germs and bone‐like structures formed by the 5OA/EPI + MES assembloid group contained newly developed CD31‐positive blood vessels, as well as OCN‐positive dentin or bone‐like matrices. In contrast, barely any blood vessels or collagen fiber formation was detected in the EPI + MES cell suspension group.

The development of the tooth requires reciprocal interactions between the dental epithelium and mesenchyme, mediated by signaling pathways such as Wingless/Int‐1, Sonic Hedgehog, bone morphogenetic protein (BMP), fibroblast growth factor.^[^ ^67^, ^68^ ^]^ Therefore, preventing the mingling of dental epithelial and mesenchymal cells is not only important for the formation of tooth structures but also necessary for precisely controlled signaling exchanges between epithelium and mesenchyme. In this study, we demonstrated that while the EPI‐Germ and MES‐Germ fused together, the two cell types remained compartmentalized in the assembloids, enabling the EMIs that drive the odontogenic program. This system is also suitable for bioengineering organs that involve EMIs, such as salivary glands.^[^ ^69^ ^]^

In vivo transplantation experiments highlight another advantage of our system for fabricating bioengineered tooth germs: its ease of transplantation. Without support from a scaffold, bioengineered tooth germs generated from cell aggregates have very little mechanical strength. Recent research has aimed to induce dental tissue organization within hydrogels in vitro.^[^ ^70^ ^]^ Ikeda et al. had to culture the cell aggregates in a collagen gel scaffold for 5–7 days to strengthen the tooth germ before dissolving the collagen gel and performing the transplantation.^[^ ^62^ ^]^ In contrast, bioengineered tooth germs fabricated using our system can, in theory, be directly transplanted, as the OA microgel provides mechanical support to the cell aggregates during the early phase of transplantation and degrades when the tooth germ needs to integrate with host tissue.

Furthermore, an addition advantage of the OA microgel in fabricating bioengineered tooth germ lies in its ability to facilitate cell condensation. Mesenchymal cell condensation is a critical step in odontogenesis in vivo, where mechanical compression of dental mesenchymal cells leads to the upregulation of critical genes such as *Pax9*, *Msx1*, and *Bmp4*.^[^ ^71^, ^72^ ^]^ However, the current bioengineered tooth germ created using our system still lacks the characteristic morphological features of the real tooth germ, especially the EPI‐Germ compartment failed to develop an epithelium‐like tissue architecture. With future optimization of the system, such as adjusting cell density and hydrogel degradation speed, we expect to create a bioengineered tooth germ that more closely resembles a natural tooth and, eventually, achieve a fully calcified tooth with a crown, root, and periodontium after transplantation into the jaw.

## 3 Conclusion

In summary, we successfully prepared 5OA microgels that ensured optimal encapsulation of high‐density hDSCs. Our in vitro modeling demonstrated effective self‐degradation of 5OA, facilitating rapid cell condensation and integration into robust 3D tissue structures. In vivo transplantation studies of both premade and immediately transplanted groups revealed significant tissue integration, with no residual OA detected, underscoring the advantages of 5OA microgels for instant injection for dental tissue remodeling. Crucially, in subcutaneous mouse models, 5OA‐based constructs facilitated rapid host tissue integration, vascular infiltration, and osteogenic differentiation showing promising translative potential. Furthermore, we reconstituted a physiologically relevant in vitro model— tooth germ model—via epithelial and mesenchymal cell coculture within 5OA microgels, which preserved cell identity, partially mimicked the natural developmental processes of teeth, supported the expression of key developmental genes (*Pitx2*, *Msx1*, and *Pax9*), and promoted mineralized tissue formation in vivo. This innovative approach not only streamlines the fabrication process, making it cost‐effective and scalable, but also opens new avenues for high‐throughput pharmacological screenings. Our findings highlight the broad applicability of this OA microgel platform as an efficient, scaffold‐free strategy for enabling functional dental tissue regeneration. The ability to achieve rapid cell condensation, coupled with enhanced vascularization and tissue remodeling, positions this system as a promising candidate for future clinical applications in regenerative dentistry and beyond.

## 4 Experimental Section

Detailed descriptions of the preparation and optimization of OA, microgel fabrication methods, and both in vitro and in vivo assessments can be found in the Supporting Information.

## Supporting Information

Supporting Information is available from the Wiley Online Library or from the author.

## Conflict of Interest

The authors declare no conflict of interest.

## Supporting information

Supplementary Material

## Data Availability

The data that support the findings of this study are available from the corresponding author upon reasonable request.
