# Supplementary material for: Harnessing Oxidized Alginate Microgels for Rapid and Self‐Assembling Dental Tissue Organogenesis In Vitro and In Vivo
Source: Small Sci. 2025 Oct 3;5(12):e202500053. doi: 10.1002/smsc.202500053 (PMC12697820; doi:10.1002/smsc.202500053)
Supplement: Supplementary file 1 — Supplementary Material [file SMSC-5-e202500053-s001.pdf]

Supporting Information

Harnessing oxidized alginate microgels for rapid and self-assembling dental tissue organogenesis *in vitro* and *in vivo*

Chao Liang[a,1], Shuxuan Wu[b,1], Ziqi Huang[a], Zhenzhen Wu[a], Siyuan Chen[a], Feiming Li[a], Karrie Mei-Yee Kiang[c], Gilberto Ka-Kit Leung[c,d], Indong Jun[e], Hwan D. Kim[f], Ann-Na Cho[g], Hee Jung Lee[h], Honghyun Park[i], Yiu Yan Leung[j], Seong Jun Kim[k], Seil Sohn[k], Haram Nah[l], Jae Seo Lee[l], Il Keun Kwon[l], Dong-Nyoung Heo[m], Sang-woo Lee[n], Zhaoming Wu[b,2,*], Sang Jin Lee[a,2,*]

[a]*Biofunctional Materials, Division of Applied Oral Sciences and Community Dental Care, Faculty of Dentistry, The University of Hong Kong, 34 Hospital Road, Sai Ying Pun, Hong Kong SAR, PRC*

[b]*Craniofacial Development, Division of Applied Oral Sciences and Community Dental Care, Faculty of Dentistry, The University of Hong Kong, 34 Hospital Road, Sai Ying Pun, Hong Kong SAR, PRC*

[c]*Department of Surgery, School of Clinical Medicine, LKS Faculty of Medicine, The University of Hong Kong, Hong Kong, 21 Sassoon Rd, Pokfulam, Hong Kong SAR, PRC*

[d]*The State Key Laboratory of Brain and Cognitive Sciences, The University of Hong Kong, Pokfulam, Hong Kong SAR, PRC*

[e]*Environmental Safety Group, Korea Institute of Science & Technology Europe (KIST-EUROPE), 66123, Saarbrücken, Germany*

[f]*Department of Biomedical Sciences, Seoul National University of Science and Technology, Seoul 01811, Republic of Korea*

[g]*School of Biomedical Engineering, Faculty of Engineering, The University of Sydney, Darlington, NSW 2008, Australia*

[h]*Composites Research Division, Korea Institute of Materials Science, 797 Changwon-daero, Seongasna-gu, Changwon, South Korea*

[i]*Advanced Bio and Healthcare Materials Research Division, Korea Institute of Materials Science, 797 Changwon-daero, Seongasna-gu, Changwon, South Korea*

[j]*Oral and Maxillofacial Surgery, Faculty of Dentistry, The University of Hong Kong, 34 Hospital Road, Sai Ying Pun, Hong Kong SAR, PRC*

[k]*Department of Neurosurgery, CHA Bundang Medical Center, CHA University, Seongnam-si, Gyeonggi-do, Republic of Korea*

[l]*Department of Dental Materials, School of Dentistry, Kyung Hee University, 26 Kyungheedae-ro, Dongdaemun-gu, Seoul, 02447, South Korea*

[m]*Biofriends Inc, 26 Kyungheedae-Ro, Dongdaemun-Gu, Seoul, 02447, South Korea*

[n]*Department of Physiology, School of Dentistry and Dental Research Institute, Seoul National University, Seoul 08826, Republic of Korea*

2. Materials & methods

2.1 Materials

Sodium alginate (Cat. No. A2033), MES hydrate (Cat. No. M8250, ≥99.5% (titration)), calcium chloride ($CaCl_2$, 33.294 g, Cat. No. C5670), sodium periodate ($NaIO_4$, Cat. No. 311448, ACS reagent, ≥99.8%), NaCl (17.53 g, Cat. No. S9888, ACS reagent, ≥99.0%), ethylene glycol (Cat. No. 102466), D_2O (Cat. No. 450510), and Tween® 20 (Cat. No. P7949) were purchased from Sigma-Aldrich (St. Louis, MO, USA). Dialysis membranes (Spectra/Por® biotech cellulose ester dialysis membranes, 3,500 MWCO) were obtained from Spectrum Laboratories, Inc. (Rancho Dominguez, CA, USA). Steritop® filters (Cat. No. SCGPS05RE) were purchased from Millipore GP Express (Billerica, MA, USA).

2.2 Preparation of OA and microgels

The 1OA, 2OA and 5OA were synthesized through an oxidation process as described in previous work, with some modifications.[1-2] Briefly, medium viscosity sodium alginate (10 g) was dissolved in 900 ml of ultrapure deionized water (diH_2O) overnight for each condition. Sodium periodate ($NaIO_4$, 0.108 g for 1OA, 0.216 g for 2OA, and 0.540 g for 5OA) was dissolved separately in 100 ml of diH_2O and then added to the alginate solution to achieve a theoretical oxidation degree of 1, 2, and 5%, respectively. This mixture was stirred vigorously in the dark (covered with aluminum foil) at room temperature for 24 hours. Subsequently, MES hydrate (19.52 g) and NaCl (17.53 g) were added directly to the oxidized alginate solution (1 L), and the pH was adjusted to 6.5. Ethylene glycol (1 mL) was then added to halt the oxidation process. The OA macromer solution was purified using a dialysis membrane in diH_2O for 5 days, with fresh diH_2O being replaced twice daily. After dialysis, the OA macromer solution was filtered through bacteria-excluding Steritop® filters with a pore size of 0.22 µm under vacuum in a cell culture hood. Finally, the products were lyophilized for at

least 10 days and stored at 4 °C until use.

For microgel generation, a 2% or 4% suspension of 1OA/2OA/5OA macromer in PBS was dropped directly into a bath containing the pre-made $CaCl_2$ crosslinker to form spheroidal microgels. To maintain sterility, this process was performed under a laminar flow hood. Similarly, the control group was prepared using a 1% or 2% UA suspension in PBS.

2.3. Preparation of $CaCl_2$ cross-linker

A sterilized $CaCl_2$ solution was used as a crosslinker for the OA/UA microgels. Briefly, 25 mM MES hydrate (9.762 g) was completely dissolved in 2 L of deionized water (diH_2O). Subsequently, 150 mM $CaCl_2$ (33.294 g) was added to the mixture, and the pH was adjusted to 7.2. The mixture was then filtered using bacteria-excluding 0.22 µm pore size Steritop® filters. Finally, 0.05 wt % Tween® 20 was added to the mixture . The prepared crosslinker was stored at room temperature until use.

For microgel generation, 0.05 wt % Tween® 20 was used to decrease the surface tension and minimize air introduction during cell encapsulation.[3] Spherical beads are obtained when the kinetic energy is high enough to break the surface resistance of the calcium bath and droplet viscosity high enough to avoid deformations. Since the increase of oxidation of OA hydrogel result in the decrease of viscosity of OA and the kinetic energy is simultaneously decrease.[3]

2.4 Characterization of oxidation and degradation of OA

To confirm the successful synthesis of OA, 20 mg of UA, 1OA, 2OA and 5OA was dissolved in 1 ml of D_2O), a ^{1}H-Nuclear Magnetic Resonance (^{1}H-NMR) spectrometer (Bruker Avance 400, Bruker Corporation, Billerica, MA, USA) was used to measure the chemical structure of OA at 25 °C.

Steady shear rheological measurements were performed using a Thermo Scientific HAAKE Rotation Rheometer (MARS™ 40) equipped with Peltier temperature control (±0.1°C). An 8 mm parallel plate (Model. No. 222-2473) (gap height: 0.3 mm), an 8 mm P8/Ti Rotor (Model. No. 01231179). OA macromer solutions (1%, 2%, 4% w/v in $CaCl_2$-free PBS) exhibiting shear-thinning behavior were prepared by 24-hour hydration at 25°C. Samples (120 μL volume) were subjected to continuous shear rate sweeps (0.1-10 s^{-1}, linear progression) over 120 s at 25.0 ± 0.1°C (n=3 independent batches). The RheoWin software (v4) automatically recorded shear stress (τ) and calculated apparent viscosity (η) in real-time using the constitutive relationship $\eta(\dot{\gamma})=\tau(\dot{\gamma})/\dot{\gamma}$. System calibration was verified using NIST S20 standard fluid (<3% deviation tolerance), while evaporation was controlled by silicone oil barriers and gap integrity maintained through continuous normal force monitoring (<0.01 N threshold).[4]

For the degradation test of UA, 1OA, 2OA and 5OA, 2% UA, 4% 1OA, 4% 2OA and 4% 5 OA were fabricated using same crosslinking method as mentioned before. 200uL of microgel were used to prepare around 40 microgel for each sample. Each sample was placed in 15mL cornical tube in 15mL Calcium ion/magnesium ion-free PBS. PBS were changed every day with 1mL remained in tubes, samples were washed with Ultra pure water for three times, lypholized and measured the weight at 1, 3, 5 days. (n=3).

For the morphology verification of the degradation of UA and OA, 1% UA microgels and 2% OA microgels were randomly selected and placed in a U-bottom, non-binding 96 well plate (Cat. No. 650901, Greiner Bio-One, Kremsmünster, Austria). The microgels were then incubated in PBS for 5 days at 5% CO_2 and 37 °C. Additionally, 1% UA microgels were incubated as a control. Bright-field images were captured on days 0, 1, 2, 3, and 5 post-incubations. Samples collected on days 0 and 5 were preserved for future paraffin sectioning.

2.5 Isolation of hDSCs and expansion

2.5.1 Institutional Review Board (IRB) approval

Human teeth were collected with the consent of the patients at the Prince Philip Dental Hospital (PPDH, Sai Ying Pun, Hong Kong). The study was conducted in accordance with the Declaration of Helsinki, and the protocol (HKWC-2024-055) was approved and supervised by the IRB of The University of Hong Kong / Hospital Authority Hong Kong West Cluster (HKU/HA HKW IRB).

2.5.2 Isolation and expansion of SCAP

SCAP were isolated from the developing apical papilla tissue of extracted human immature third molars, which exhibited radiographic evidence of an immature developmental stage with open apices (>1.5 mm). All donors, aged 13 to 25 years (or their parent/guardian when the patient was under 18 years), provided informed consent for inclusion in the study prior to participation. The isolated tissue was digested with 3 mg/mL collagenase type I (Cat. No. BS-163, Biosharp, Anhui, China) at 37 °C for 30 minutes. SCAP were cultured in Minimum Essential Medium Eagle alpha modification (αMEM; Cat. No. 11900-073, Gibco, Waltham, MA, USA) supplemented with 10% fetal bovine serum (FBS) (Cat. No. A5256701, Gibco, Waltham, MA, USA), 100 U/mL penicillin, and 100 μg/mL streptomycin (Cat. No. 151400-122, Gibco, Waltham, MA, USA) under conditions of 5% CO_2 and 37 °C. After 7 to 10 days of culture, cells were observed around the tissue blocks. Once the cells reached 80% to 90% confluence, they were digested with 0.25% Trypsin-EDTA (Cat. No. 25200-072, Gibco, Waltham, MA, USA) and transferred to a new dish for further expansion. The medium was changed every 2 to 3 days. SCAP at the third passage were used for subsequent experiments.

2.5.3 Isolation and expansion of PDLSC

For the primary culture of PDLSC, premolars and impacted third molars were collected from healthy donors aged 20 to 40 years. Periodontal ligament tissue was scraped from the middle third of the roots, washed with PBS, and collected by centrifugation. The isolated tissue was digested with 3 mg/mL collagenase type I (Biosharp, Anhui, China) at 37 °C for 30 minutes. The digestion process was terminated by adding an equal volume of culture medium. The tissue fragments were isolated by centrifugation and transferred into a culture plate under the same conditions used for SCAP described in section 2.6.1. Cells were incubated at 5% CO_2 and 37 °C. After 3 to 8 days of culture, cells were observed around the tissue blocks. When the cells reached 80% to 90% confluence, they were digested with 0.25% Trypsin-EDTA and transferred for further expansion. The passaged cells were cultured in a new Dulbecco's Modified Eagle Medium (DMEM) (Cat. No. 12100046, Gibco, Waltham, MA, USA) containing 10% fetal bovine serum (FBS) (Cat. No. A5256701, Gibco, Waltham, MA, USA), 100 U/mL penicillin, and 100 µg/mL streptomycin (Cat. No. 151400-122, Gibco, Waltham, MA, USA). The medium was changed every 2 to 3 days. PDLSC at the third passage were used for subsequent experiments.

2.6 Optimization of OA and high-density cell encapsulation

2.6.1 Optimization of oxidation of OA microgel for high-density cell encapsulation

OA microgels at different oxidation degrees (1OA, 2OA, 5OA; 2% w/v) and unmodified alginate (UA; 1% w/v control) were prepared and encapsulated immortalization of human mesenchymal stem cells by human telomerase reverse transcriptase (hMSCs-TERT) kindly provided by Dr. Dario Campana group.[5] The hMSCs-TERT (1×10^8 cells/mL) contained in UA or OA macromer solutions were dropped into pre-gelled $CaCl_2$ crosslinker to form

spherical microgels.

Cell quantification: A single microgel droplet (around 5 μL) was dispersed in 1 mL of PBS. Encapsulated cells were counted manually using a hemocytometer (n = 3). Roundness were calculated using NIH ImageJ. Microgel volume estimation: Microgels formed from 1 mL of polymer solution were counted after crosslinking in $CaCl_2$. Average microgel volume was calculated as: Volume (μL/microgel) = 1000 μL / total microgel count. Bright-field imaging of single and double hMSC-TERT-laden microgel constructs was performed on days 0, 1, 2, 3, 5, and 7. Cell only groups containing 2 x 10^5 and 4 x 10^5 cells per well served as a positive control for single and double microgel models. Diameters and overlapping areas were quantified using ImageJ software (n > 25 per group). Cellular viability within microgels was assessed through Live/Dead fluorescence imaging after stained by Live/Dead cell labeling kit (Cat. No. C2015M, Beyotime, Shanghai, China), with calcein-AM (ex/em 495/515 nm) and propidium iodide (ex/em 535/617 nm) channels acquired separately using standardized parameters. Microgel regions of interest were delineated using bright-field reference images, after which fluorescence intensity quantification was performed in Fiji/ImageJ (v2.16). Background correction was applied by subtracting mean intensity values measured in acellular hydrogel regions from corresponding live and dead channel measurements. The viability index was subsequently calculated as the ratio of background-corrected live channel intensity to the sum of background-corrected live and dead channel intensities, expressed mathematically as:

$$I_{\mathrm{corr}} = I_{\mathrm{sample}} - I_{\mathrm{background}}$$

where background measurements were obtained from acellular hydrogel regions. The dying index (DI) was calculated as:

$$DI = \mathrm{I}_{PI\text{-}corr} / (\mathrm{I}_{calcein\text{-}corr} + \mathrm{I}_{PI\text{-}corr})$$

2.6.2 Fabrication of high-density SCAP-laden OA microgels

The required number of SCAP was prepared using the standard cell culture expansion method described in section 2.5.2. The obtained cell pellets at a density of 1.5 x 10^8 were mixed directly with 1 ml of 2% OA in PBS. After vigorous mixing, the OA macromer suspensions containing SCAP were dropped into the pre-made $CaCl_2$ cross-linker to form spheroidal OA microgels. Similarly, the control group was prepared using 1 ml of 1% UA in PBS with SCAP at a density of 1 x 10^8.

2.7 *In vitro* condensation and integration of OA/SCAP and UA/SCAP microgels

To demonstrate cell condensation within microgels, single SCAP-laden OA microgels (OA/SCAP) were randomly selected and placed individually in each well of a clear, U-bottom, non-binding 96-well plate (Cat. No. 650901, Greiner Bio-One, Kremsmünster, Austria), while SCAP-laden UA microgels were used as a control. For the demonstration of tissue integration between microgels, two OA/SCAP microgels were placed in one well of a U-bottom 96-well plate, alongside two UA/SCAP microgels as control. Bright-field images of each group were captured on days 0, 1, 2, 3, and 5. Live/dead staining was performed on day 5 using a Hoechst 33342/PI dual staining kit (Cat. No. BL116A, Biosharp, Anhui, China). Samples from days 0 and 5 were preserved in 10% normal buffered formalin (NBF) followed by 70% ethanol at 4 °C for paraffin sectioning.

For paraffin sectioning, samples were dehydrated using gradient ethanol and xylene, embedded in paraffin, and sectioned at a thickness of 7 μm using a rotary microtome (Leica RM2155, Leica Microsystems, Wetzlar, Germany). Sections were deparaffinized and rehydrated prior to histological staining. They were stained with 0.5% safranin O (Cat. No. 02782-25, Polysciences Inc., Warrington, PA, USA) with fast green (Cat. No. PH1852, Scientific Phygene®) as a counterstain, and hematoxylin (Cat. No. SH4777, Harris

Hematoxylin, Cancer Diagnostic Inc., Durham, NC, USA) and eosin (Cat. No. CS701, Dako, Glostrup, Denmark) for H&E staining.

2.8 Animal care

All animal studies were conducted under the animal research protocol (No. 22-268 for subcutaneous implantation and No. 25-057 for kidney implantation) approved by the Committee on the Use of Live Animals in Teaching and Research (CULATR) at the University of Hong Kong. The studies adhered to the Animals (Control of Experiments) Ordinance (Hong Kong) and guidelines from the Centre for Comparative Medical Research (CCMR), Li Ka Shing Faculty of Medicine, The University of Hong Kong.

The NOD. Cg-Prkdc scid Il2rg tm1Wjl /SzJ mouse strain, commonly known as NSG mice (6-8 weeks old), was used for the experiments. The mice were obtained from the Experimental Animal Center of the University and had access to food and water ad libitum, being maintained under pathogen-free conditions. All procedures were performed under anesthesia using intraperitoneal injections of a ketamine/xylazine mixture (100 mg/kg ketamine [Cat. No. 013004, AlfaMedic Ltd.] + 10 mg/kg xylazine [Cat. No. 013006, AlfaMedic Ltd.]). Anesthesia was confirmed by checking body reflexes, and eye ointment (Duratears® ointment, Cat. No. 05686, Alcon, Fort Worth, TX, USA) was applied to prevent blindness. The subcutaneous transplantation sites on the mice were decontaminated with alternating applications of povidone-iodine (Betadine®) and 70% alcohol using sterile cotton swabs. The animals administered buprenorphine (~0.1 mg/kg) 30-60 minutes before surgery and twice a day for three days thereafter.

2.9. *In vivo* transplantation of OA/PDLSC microgels and UA/PDLSC microgels

2.9.1 Subcutaneious transplantation of pre-cultured and freshed UA/PDLSC microgels

and OA/PDLSC microgels for 2 days *in vivo*

To confirm the release of cells from OA microgels and the integration of the transplant with host tissue, *in vivo* transplantation was performed using UA/PDLSC and OA/PDLSC. The PDLSC pellets were prepared as described in section 2.5.3, followed by the encapsulation of PDLSC in UA/PDLSC and OA/PDLSC using the same procedures outlined above. To assess the immediate transplantation potential, we transplanted the 2-day precultured *in vitro* UA/PDLSC and OA/PDLSC groups or the immediately crosslinked UA/PDLSC and OA/PDLSC groups, respectively. Before transplantation, the dorsal subcutaneous area was divided into four quadrants and the transplantation was performed. The four quadrants were assigned to four groups as follows: **i)** 2D precultured UA/PDLSC (left upper); **ii)** 0D UA/PDLSC (immediate transplantation, right upper); **iii)** 2D pre-cultured OA/PDLSC (left lower); **iv)** 0D OA/PDLSC (immediate transplantation, right lower).

Microgels were transplanted into the backs of the mice with volumes of ~250 μL per site (Figure 1). After suturing, the mice were placed in an intensive care unit (ICU) until they fully recovered, after which they were returned to their original housing location. 2 days post-implantation, the mice were euthanized with an overdose of pentobarbital (250 mg/kg) (Dorminal®, Cat. No. 013003, AlfaMedic Ltd.), administered intraperitoneally. Samples and tissues were harvested. Transplants were fixed in 10% NBF for 24 hours for histological analysis, while day 0 samples were kept in 70% ethanol as a control.

2.9.2 Subcutaneous transplantation of fresh 5OA/PDLSC microgels and UA/PDLSC microgels for 3 week *in vivo*

Fresh UA/PDLSC and 5OA/PDLSC microgels were fabricated as described in Sections 2.4 and 2.5.3. Approximately 200 μL of each microgel type was transplanted into the left or right dorsal subcutaneous area of 6–8-week-old NSG mice. Surgical procedures (aseptic

technique), postoperative care, and monitoring followed Section 2.9.1. At 1, 2, and 3 weeks post-transplantation, mice were euthanized via pentobarbital overdose (250 mg/kg, i.p.; Dorminal®, AlfaMedic Ltd., Cat. No. 013003), and transplants were harvested for analysis.

2.9.3 Subcutaneous transplantation of osteogenic 2 weeks' pre-cultured 5OA/PDLSC microgels and UA/PDLSC microgels

UA/PDLSC and OA/PDLSC microgels were prepared per Sections 2.5.3 and 2.4, then cultured in osteogenic medium[6] for 2 week prior to transplantation. Approximately 200 μL of each pre-differentiated microgel was transplanted into the left or right dorsal subcutaneous area of NSG mice. Identical surgical protocols, postoperative care, and euthanasia methods (pentobarbital overdose, 250 mg/kg, i.p.) were applied as in Section 2.9.1. Transplants were harvested 1 week post-surgery for analysis.

2.9.4 Tissue processing

A stereoscope was used to acquire gross images of the harvested samples after fixation in 70% ethanol. All samples were dehydrated using a gradient of ethanol (from 70% to 100%) and underwent a series of treatments with ethanol, xylene-ethanol, and xylene, each lasting 30 minutes. Subsequently, all samples were embedded in paraffin and sectioned to a thickness of 7 μm using a rotary microtome (Cat. No. Leica RM2155, Leica Microsystems, Wetzlar, Germany). The sections were deparaffinized, rehydrated, and stained with hematoxylin (Cat. No. SH4777, Harris Hematoxylin, Cancer Diagnostic Inc., Durham, NC, USA) and eosin (Cat. No. CS701, Dako, Glostrup, Denmark) (H&E), as well as with 0.5% safranin O (Cat. No. 02782-25, Polysciences Inc., Warrington, PA, USA) combined with fast green (Cat. No. PH1852, Scientific Phygene®, Fuzhou, China) and alcian blue (pH 2.5) with 0.1% nuclear fast red (Cat. No. PH1082, Scientific Phygene®, Fuzhou, China).

Immunofluorescence staining for hHNA, CD31, OCN, and Runx2 was performed as follows: After deparaffinization and rehydration, slides were permeabilized/blocked with 0.1% Triton X-100 (Cat No. ST023, Sigma-Aldrich, St. Louis, MO, USA) / 0.1% Tween-20 (Cat No. 85115, Sigma-Aldrich, St. Louis, MO, USA) / 3% BSA (Cat No. 85112, Beyotime, Shanghai, China) for 1 h at room temperature. Primary antibodies incubated overnight at 4°C included: goat anti-CD31 (Cat No. AF3628; R&D Systems, Minneapolis, MN, USA), rabbit anti-OCN (Cat No. PA5-96529; Invitrogen, Carlsbad, CA, USA), rabbit anti-Runx2 (Cat No. 12556; Cell Signaling Technology, Danvers, MA, USS), and rabbit anti-hHNA (Cat No. NBP3-13912; Novus Biologicals, Centennial, CO, USA). After PBS washes (3 × 5 min), secondary antibodies were applied for 1 h at room temperature one by one: donkey anti-goat IgG-Alexa Fluor 488 (Cat No. A-11055; Invitrogen, Carlsbad, CA, USA) and washed with PBS washes (3 × 5 min), then treated with goat anti-rabbit IgG-Alexa Fluor 594 (Cat No. A-11012; Invitrogen, Carlsbad, CA, USA). Nuclei were counterstained with 10 μg/mL DAPI (Cat No. C1006; Beyotime, Shanghai, China) for 5 min, followed by final PBS washes (3 × 5 min), and slides were mounted with antifade medium (Cat No. PH0428; Phygene, Fuzhou, China). Quantification of OCN-positive and CD31-posive were done by Image J.

2.10 Generation of tooth germ model and EPI-Mes tissue crosstalk

2.10.1 Animals for tooth-germ extraction

Time-mated pregnant CD1 mice were purchased from the Centre of Comparative Medical Research at The University of Hong Kong. The pregnant mice were euthanized via an overdose of sodium pentobarbital anesthesia (150mg/ml) to collect the mouse embryos. The experiments were conducted under the animal research protocol (No. 6059-22) approved by the CULATR at the University of Hong Kong.

2.10.2 Tooth germ isolation and reconstitution *in vitro*

The first molar tooth germs were isolated from the embryonic lower jaw in cold PBS under a stereo microscope. The tooth germs were treated with dispase II (1.3 U/ml, Roche, Basel, Switzerland) at 37 °C for 30 minutes to separate the dental epithelium from the dental mesenchyme. Part of the isolated dental epithelium and mesenchyme was collected for qRT-PCR analysis. Single-cell suspensions of dental epithelium and dental mesenchyme were prepared by treating the tissues with 0.25% Trypsin-EDTA solution for 3 minutes, using micropipette titration. The epithelial cells and mesenchymal cells were counted and suspended in 2% OA macromer solution prepared by PBS at cell densities of 2.16 x 10^7 and 4.66 x 10^7 / ml respectively. The densities were determined by the cell yield from embryonic tooth germs, reflecting the natural epithelial-to-mesenchymal cell ratio in the developing tooth germ and allowing appropriate cell-cell contact and epithelial-mesenchymal interactions. To promote cell aggregation and facilitate spatial organization, a V-bottom, non-binding 96-well plate (Cat. No. 651301, Greiner Bio-One, Kremsmünster, Austria) was employed. Each well contained one epithelial bead and one mesenchymal bead to enable their spontaneous fusion. Each well of the microplate was filled with 100 µL DMEM (Cat. No. 12100046, Gibco, Waltham, MA, USA) supplemented with 10% FBS (Cat. No. A5256701, Gibco, Waltham, MA, USA)and 1x GlutaMax (Cat. No. 35050061, Gibco, Waltham, MA, USA).

2.10.3 Tooth germ cultivation

The same method was used to fabricate the tooth germ model: epithelial stem cells and mesenchymal stem cells isolated from mice at embryonic day (ED) 14.5 were separately encapsulated within OA microgels to form epithelial spheroids (EPI-Germ) and mesenchymal spheroids (MES-Germ). To visualize the fusion process, epithelial and mesenchymal cells were labelled with DiO (Cat. No. V22886, Invitrogen, Carlsbad, CA, USA) and DiI (Cat. No.

V22885, Invitrogen, Carlsbad, CA, USA), respectively, before being mixed together. Epithelial and mesenchymal cell suspensions were cultured either individually or mixed together as controls. Morphological changes were monitored daily from day 0 to day 7 in all groups. In addition, samples were collected for RNA extraction to assess the expression levels of relevant genes by qRT-PCR using the StepOnePlus™ Real-Time PCR system (Cat No. 43-766-00, Thermo Scientific, Waltham, MA, USA).

For qRT-PCR, total RNAs were extracted from cells using Quick-RNA Microprep Kit (Cat. No.R1050, Zymo Research, Irvine, CA, USA) and then reverse-transcribed to cDNA with the FAST RT Reagent Kit (Cat. No. RR092a, Takara, Osaka, Japan) according to the manufacturer's protocol. The quantitative PCR was performed using TB Green Premix Ex Taq™ II (Cat. No. CN830S, Takara, Osaka, Japan), and the relative expression levels were calculated with the comparative threshold cycle (ΔΔCT) method. All tests were performed in triplicate. Primers sequences are as Msx1: Forward: TCTCGGCCATTTCTCAGTCG; Reverse: CCGATCTAGTTTCTCGGGGC. Pitx2 Forward: TCCTCACCCTTCTGTCACCAT; Reverse: GGCCCTTATCTTTCTCTGCGA.

The microstructure of the epithelial-mesenchymal aggregates was evaluated through cryo-sectioning and histological staining. Samples on day 4 were preserved for cryo-sectioning. The specimens were preserved using liquid nitrogen freezing and then embedded in OCT (Cat. No. 4583, Sakura Finetek, Torrance, CA, USA) before being sliced into 7 μm thick sections using a frozen section microtome (Leica CM1860 UV, Leica Microsystems, Wetzlar, Germany). Sections were washed of OCT and rehydrated prior to histological staining. They were subsequently processed with hematoxylin and eosin (H&E), *in situ* hybridization (ISH), and immunofluorescence (IF).

For ISH, a Pax9 RNA probe was designed based on its mRNA sequences. The plasmid template of the mouse Pax9 probe was amplified by PCR and cloned into the PCRII vector

using the following primers: Pax9 F: TTACTGCCTCAAGAGCTGGC, R: CACCAGCAAGTCACCTTTGC. The RNA probe was synthesized through transcription and RNA precipitation. ISH was performed on frozen sections, which were thawed at room temperature for 30 minutes and washed three times with PBST. The sections were then permeabilized with Proteinase K (Cat. No. 9034, Takara, Osaka, Japan) for 5 minutes at room temperature, followed by post-fixation in 4% paraformaldehyde (PFA) for 20 minutes. After several washes in PBST, the sections were incubated in 70% and 95% ethanol sequentially and air-dried in an open humid chamber. Hybridization was carried out overnight with digoxigenin-labeled (Cat. No.11277073910, Roche, Basel, Switzerland) Pax9 RNA probes at 65°C. Following several stringent washes, the sections were blocked with 10% goat serum in TBST (TBS/0.1% Tween) for 2 hours at room temperature in a humid chamber. Probe detection was performed using alkaline phosphatase (AP) conjugated to anti-digoxigenin Fab fragments (Cat. No. 11093274910, Sigma-Aldrich, St. Louis, MO, USA). After an overnight wash in TBST, the AP reaction was developed with 4-Nitro Blue Tetrazolium Chloride (NBT, Cat. No. 11383213001, Sigma-Aldrich, St. Louis, MO, USA) and 5-Bromo-4-chloro-3'-indolyphosphate p-toluidine salt (BCIP, Cat. No. B8503, Sigma-Aldrich, St. Louis, MO, USA), and the color reaction was stopped with PBST once optimal development was achieved. The sections were then co-stained with 0.1% nuclear fast red, mounted, and stored at 4°C in the dark for microscopic analysis.

For IF, the frozen sections were thawed for 30 minutes at room temperature and washed with PBST (PBS/0.1% Tween) three times to remove the OCT. Afterwards, the sections were blocked with 0.1% Triton X-100 / 3 % BSA / PBST at room temperature for 1 hour. The sections were then incubated with the primary polyclonal antibody, goat E-Cadherin (Cat. No. AF748, R&D, Minneapolis, MN, USA), at a concentration of 10 μg/ml at 4°C overnight. The next day, the sections were incubated for 2 hours with the secondary antibody, donkey

anti-goat IgG-AF488 (Cat. No. A-11055, Invitrogen, Carlsbad, CA, USA), at room temperature in the dark. Finally, the sections were washed with PBST three times and stained with 0.1% DAPI (Cat. No. D1306, Invitrogen , Carlsbad, CA, USA) in PBST before mounting with FluorSave™ Reagent (Cat. No. 345789, Sigma-Aldrich, MO, USA). Specimens were then stored in the dark at 4°C until microscopy analysis.

2.10.4 Renal capsule transplantation

The 5OA-encapsulated epithelial–mesenchymal tooth germs (5OA/EPI + MES assembloid), embryonic tooth germs (positive control), and EPI + MES cell suspensions were cultured overnight and transplanted into the renal capsule of 6–8-week-old NSG mice. All grafts were harvested 2 weeks after transplantation, fixed in 4% paraformaldehyde, paraffin-embedded, and sectioned for further analysis. Paraffin sectioning was performed as described above, with a section thickness of 7 μm. The sections were subjected to histological analyses, including H&E staining, Masson's trichrome staining, and immunofluorescence. For immunofluorescence, primary antibodies included goat anti-CD31 polyclonal antibody (Cat. No. AF3628, R&D, Minneapolis, MN, USA), rabbit anti-osteocalcin polyclonal antibody (Cat. No. PA5-96529, Invitrogen, Carlsbad, CA, USA), and rabbit anti-Runx2 polyclonal antibody (Cat. No. 12556, CST, Danvers, MA, USA). Secondary antibodies included donkey anti-goat IgG-AF488 (Cat. No. A-11055, Invitrogen, Carlsbad, CA, USA) and goat anti-rabbit IgG-AF594 (Cat. No. A-11012, Invitrogen, Carlsbad, CA, USA). The experimental procedures were performed as described above.

2.11 Quantification and statistical analysis

Image analysis was performed using Fiji/ImageJ software (version 2.16; National Institutes of Health, Bethesda, MD, USA). Images were converted to 8-bit grayscale and calibrated to the appropriate spatial scale using the scale bar in each image. Regions of interest (ROIs)

were segmented from the background by thresholding, and the "Analyze Particles" functions were used to quantify morphological parameters. Depending on the image set, area, diameter, roundness and cell number were measured using standard ImageJ measurement options. Diameter was reported as Feret's diameter or equivalent circular diameter, and roundness was calculated automatically by ImageJ, with a value of 1.0 representing a perfect circle.

For fluorescence intensity analysis, all images within the same experimental set were acquired using identical microscope settings. Mean fluorescence intensity was calculated as the integrated density (sum of pixel intensities) divided by the ROI area. Background correction was performed by measuring the mean intensity from three cell-free regions in each image and subtracting this value from the ROI measurements. Fluorescence profile values of E-cadherin were plotted as a function of distance across the EPI + MES assembloids region. For each position (x) along the sampling area, the grayscale values from the corresponding single column of pixels were averaged to obtain the fluorescence profile value (y), which was then used to generate the profile plot.

Measurement data were exported from the ImageJ Results table and analyzed in GraphPad Prism (version 9; GraphPad Software, San Diego, CA, USA) using Student's *t*-test or one-way ANOVA. P-value ≤ 0.05 was considered statistically significant.

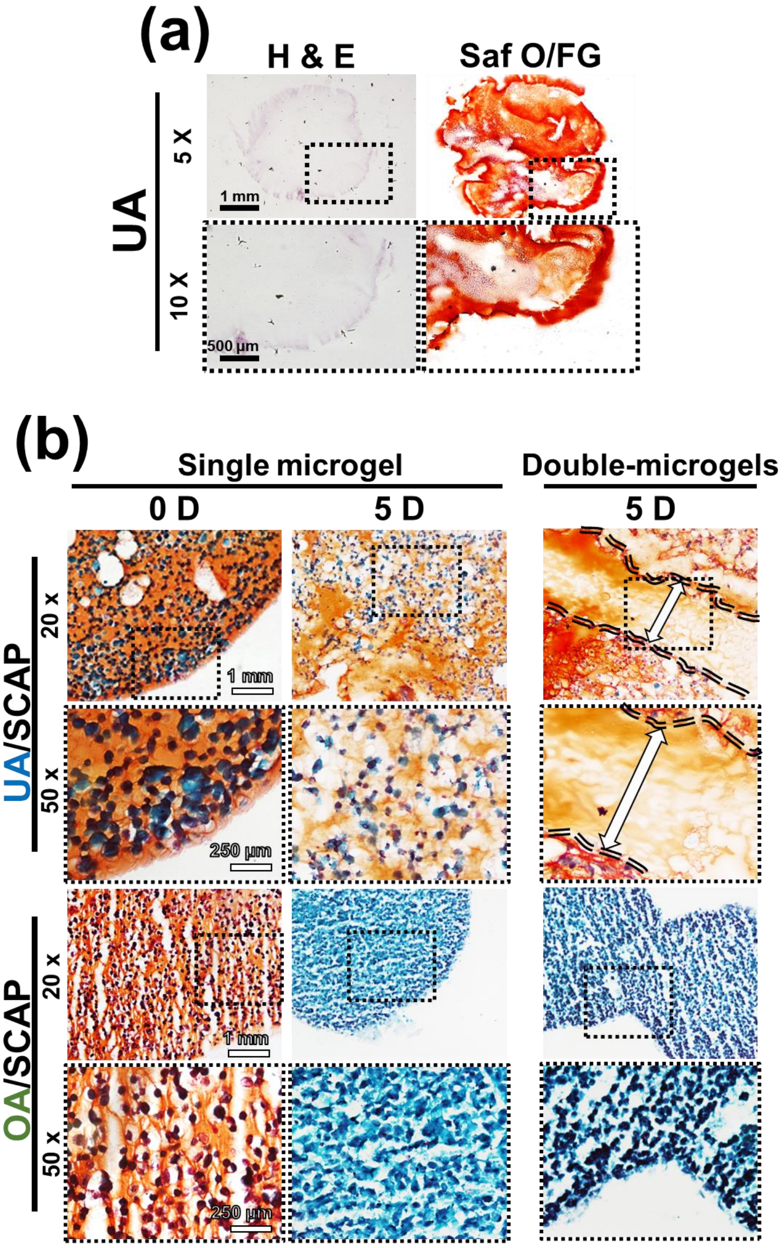

Figure S1. Histological analysis of cell-free UA microgels using H&E and safranin O with fast green staining on day 5 after culture. (a) The light purple in H&E and the strong red color in safranin O with fast green staining present the original structure of residual U. (b) The high magnification images of safranin O with fast green staining for OA/SCAP and UA/SCAP, comparing single and double particles on day 0 and day 5. Black dotted rectangles indicate zoomed-in areas, double black dashed lines represent the borders between double-microgels,

and white arrows with black edges denote the distance between the double-microgels.

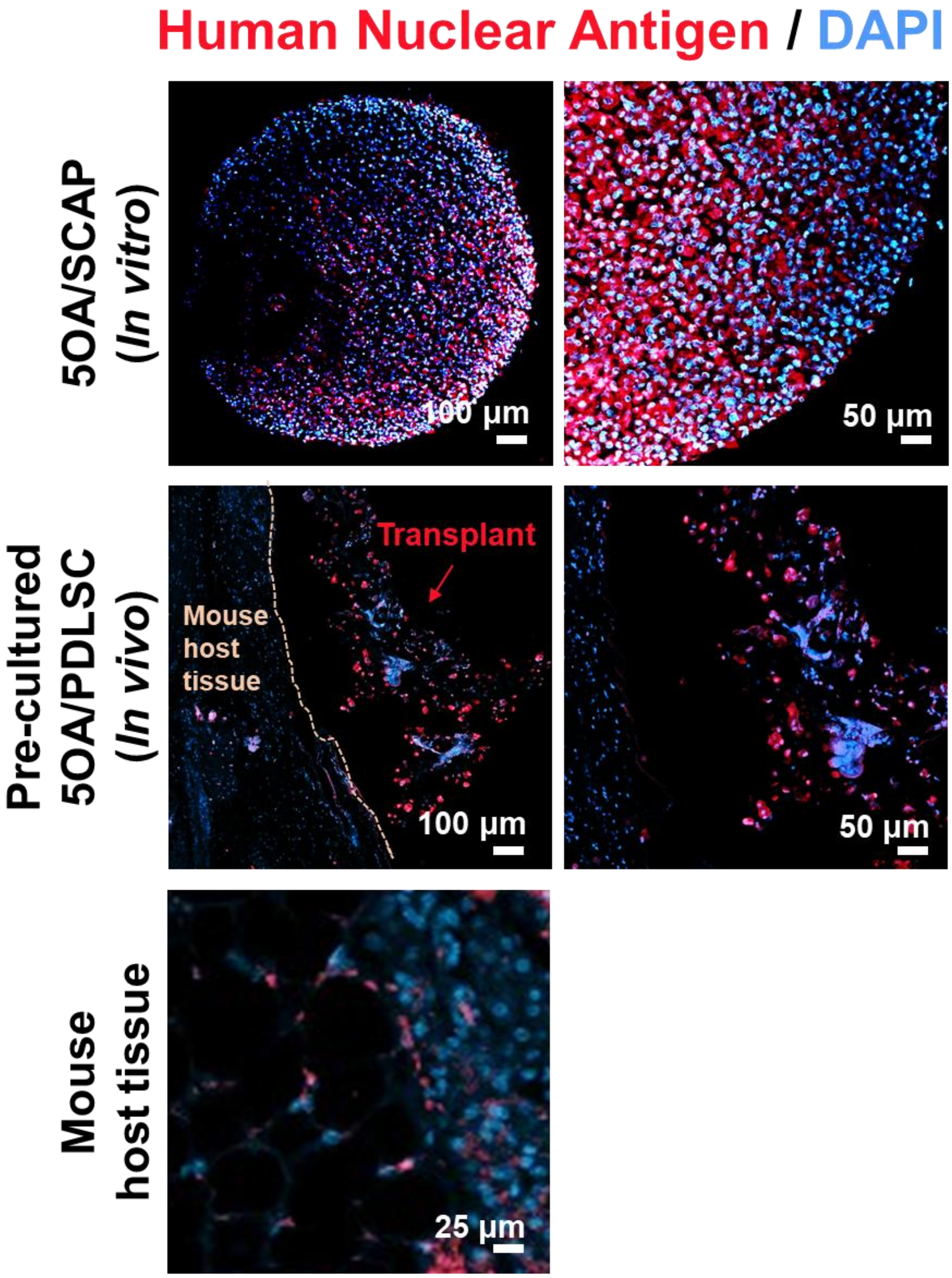

Figure S2. HNA staining of pre-cultured 5OA/PDLSC transplants at day 2. Human cells (red) were confirmed in pre-cultured 5OA/PDLSC transplants, demonstrating successful graft tissue harvest. Mouse host tissue without human cells served as a negative control, while human cells from the in vitro sample served as a positive control.

References

[1] S. J. Lee, O. Jeon, Y. B. Lee, D. S. Alt, A. Ding, R. Tang, E. Alsberg, *Advanced Composites and Hybrid Materials* **2025**, 8, 1.

[2] A. Ding, O. Jeon, D. Cleveland, K. L. Gasvoda, D. Wells, S. J. Lee, E. Alsberg, *Advanced Materials* **2022**, 34, 2109394.

[3] F. Davarcı, D. Turan, B. Ozcelik, D. Poncelet, *Food Hydrocolloids* **2017**, 62, 119.

[4] P. Bertsch, M. Diba, D. J. Mooney, S. C. Leeuwenburgh, *Chemical Reviews* **2022**, 123, 834.

[5] K. Mihara, C. Imai, E. Coustan-Smith, J. S. Dome, M. Dominici, E. Vanin, D. Campana, *British journal of haematology* **2003**, 120, 846.

[6] S. J. Lee, D. Lee, T. R. Yoon, H. K. Kim, H. H. Jo, J. S. Park, J. H. Lee, W. D. Kim, I. K. Kwon, S. A. Park, *Acta biomaterialia* **2016**, 40, 182.
